# Knock-down of *pantothenate kinase 2* severely affects the development of the nervous and vascular system in zebrafish, providing new insights into PKAN disease

**DOI:** 10.1016/j.nbd.2015.10.010

**Published:** 2016-01

**Authors:** Daniela Zizioli, Natascia Tiso, Adele Guglielmi, Claudia Saraceno, Giorgia Busolin, Roberta Giuliani, Deepak Khatri, Eugenio Monti, Giuseppe Borsani, Francesco Argenton, Dario Finazzi

**Affiliations:** a Department of Molecular and Translational Medicine, University of Brescia, viale Europa 11, 25123 Brescia, Italy; b Department of Biology, University of Padova, via U. Bassi 58/B, 35121 Padova, Italy; c Clinical Chemistry Laboratory, Spedali Civili Hospital, 25123 Brescia, Italy

**Keywords:** Pank2, NBIA, Neurodegeneration, Coenzyme A, Pantethine, Morpholino, Zebrafish

## Abstract

Pantothenate Kinase Associated Neurodegeneration (PKAN) is an autosomal recessive disorder with mutations in the pantothenate kinase 2 gene (*PANK2*), encoding an essential enzyme for Coenzyme A (CoA) biosynthesis. The molecular connection between defects in this enzyme and the neurodegenerative phenotype observed in PKAN patients is still poorly understood. We exploited the zebrafish model to study the role played by the *pank2* gene during embryonic development and get new insight into PKAN pathogenesis. The zebrafish orthologue of h*PANK2* lies on chromosome 13, is a maternal gene expressed in all development stages and, in adult animals, is highly abundant in CNS, dorsal aorta and caudal vein. The injection of a splice-inhibiting morpholino induced a clear phenotype with perturbed brain morphology and hydrocephalus; edema was present in the heart region and caudal plexus, where hemorrhages with reduction of blood circulation velocity were detected. We characterized the CNS phenotype by studying the expression pattern of *wnt1* and *neurog*1 neural markers and by use of the Tg(*neurod*:*E*GFP/*sox10*:dsRed) transgenic line. The results evidenced that downregulation of *pank2* severely impairs neuronal development, particularly in the anterior part of CNS (telencephalon). Whole-mount *in situ* hybridization analysis of the endothelial markers *cadherin-5* and *fli1a*, and use of Tg(*fli1a*:EGFP/*gata1a*:dsRed) transgenic line, confirmed the essential role of *pank2* in the formation of the vascular system. The specificity of the morpholino-induced phenotype was proved by the restoration of a normal development in a high percentage of embryos co-injected with *pank2* mRNA. Also, addition of pantethine or CoA, but not of vitamin B5, to *pank2* morpholino-injected embryos rescued the phenotype with high efficiency. The zebrafish model indicates the relevance of *pank2* activity and CoA homeostasis for normal neuronal development and functioning and provides evidence of an unsuspected role for this enzyme and its product in vascular development.

## Highlights

• Zebrafish *pank2* gene is highly expressed in the CNS and the main vascular structures.
• *Pank2* down-regulation severely affects the development of the forebrain.
• *Pank2* down-regulation affects the dorsal aorta, caudal vein and inter-somitic vessels.
• Pantethine and Coenzyme A restore the normal development in the absence of *pank2* expression.

## 1 Introduction

Pantothenate Kinase-Associated Neurodegeneration (PKAN, OMIM ID: 234200) is the most common form of a group of inherited neurological disorders named Neurodegeneration with Brain Iron Accumulation (NBIA) (Hayflick et al., 2003, Levi and Finazzi, 2014). PKAN is an autosomal recessive disease that usually manifests in early childhood with dystonia, spasticity and pigmentary retinopathy (Hayflick, 2014, Kruer et al., 2011), and often proceeds in a stepwise neuroregression with loss of motor skills, cognitive decline and premature death; in a minority of atypical cases there is a later onset and the progression is much slower (Kurian and Hayflick, 2013). A distinctive diagnostic sign is the detection of the “eye of the tiger” on T2*-weighted magnetic resonance imaging, *i.e*. a bilateral areas of hyperintensity within a region of hypointensity in the medial globus pallidus (Hayflick, 2003). A recent histological study on genetically-confirmed cases demonstrated that this brain region is severely affected, showing iron accumulation, significant neural loss and abundant spheroidal structures (Kruer et al., 2011). Disease-causing mutations were found in the Pantothenate Kinase 2 gene (*PANK2*) (Zhou et al., 2001); the encoded enzyme catalyzes the phosphorylation of pantothenate (vitamin B5), first and limiting step in the CoA biosynthesis pathway. The other PANK proteins (PANK1a, PANK1b and PANK3) share the same function in mammalians cells and tissues, but show important differences in terms of sub-cellular compartmentalization, regulation and expression. In particular human PANK2 is ubiquitous, but highly expressed in the brain (Zhou et al., 2001, Leonardi et al., 2007a), and localizes in the mitochondrial intermembrane space (Hörtnagel et al., 2003, Johnson et al., 2004, Kotzbauer et al., 2005) and possibly in the nucleus (Alfonso-Pecchio et al., 2012), whereas the other paralogs are predominantly found in the cytosol of cells (Alfonso-Pecchio et al., 2012), where most of CoA biosynthetic process happens. Furthermore, hPANK2 is particularly sensitive to feedback inhibition by acetyl-CoA (IC_50_ = 0.1 μM), but can be activated by long-chain acyl-carnitines (Leonardi et al., 2007b, Vallari et al., 1987). On the basis of these features it was suggested that it may function as a sensor for mitochondrial CoA requirement and regulator of CoA biosynthesis. The discovery that many PANK2 mutants show normal catalytic activity, at least *in vitro* (Kotzbauer et al., 2005, Zhang et al., 2006), challenges the simplest hypothesis of a shortage of cellular CoA, that has never been documented in patients and fibroblasts from patients or in mouse models of the disease. Nonetheless, a growing body of evidence indicates that perturbation of CoA homeostasis is the key factor in disease development, and the identification of mutations in another enzyme of this biochemical pathway, COASY, in two patients with NBIA further strengthen this notion (Dusi et al., 2014). This is particularly evident in the Drosophila model of the disease, where the reduced levels of expression of the only dPANK/Fbl enzyme results in reduced CoA levels, locomotor dysfunction and neurodegeneration with severe morphological alterations of mitochondria (Bosveld et al., 2008, Rana et al., 2010). An almost complete rescue of the phenotype observed in the hypomorph mutant *dPANK*/*fbl* is obtained by a “substitution therapy”, that is feeding animals with pantethine (Rana et al., 2010). Pantethine feeding is also effective in ameliorating the diseased phenotype observed in *Pank2*^−/−^ mice exposed to a ketogenic diet (Brunetti et al., 2014). Studies in animal and cellular models of the disease also provided important information about the cellular processes that may be affected when PANK2 function is defective. They evidenced perturbation of mitochondria morphology and functionality (Bosveld et al., 2008, Rana et al., 2010, Brunetti et al., 2014, Siudeja et al., 2011, Brunetti et al., 2012), increased oxidative stress and perturbed iron homeostasis (Poli et al., 2010, Campanella et al., 2012, Santambrogio and Dusi, 2015), alteration of tubulin and histones acetylation (Siudeja et al., 2012). Yet the molecular connection between such phenomena is poorly defined and many fundamental questions about the existing relationship among *PANK2*, CoA metabolism and neurodegeneration remain unanswered. Zebrafish possesses a single ortholog of *PANK2* together with three other paralogs (*pank1a*, *pank1b* and *pank4*) and could represent a relevant tool to study *pank2* function in different tissues and its role during embryonic development. Here we describe an in-depth morpho-functional characterization of the gene in zebrafish and provide functional insights of potential relevance for PKAN pathology in humans.

## 2 Materials and methods

### 2.1 Bioinformatic analysis

Identification of pantothenic acid kinase family members in zebrafish was initially performed by querying the Gene and HomoloGene databases at NCBI [PMID: 25398906]. More detailed genome sequence analysis were carried out using the University of California Santa Cruz (UCSC) Genome Browser (http://genome.ucsc.edu/) on the Zv9 (July 2010) *Danio rerio* assembly, with data validated also on the more recent (Sep. 2014) Zv10 release. In our bioinformatic investigations we also used the Ensembl zebrafish genome database (http://www.ensembl.org/Danio_rerio/Info/Index). Synteny analysis was achieved using both the Genomicus synteny browser [PMID: 20185404] and the Synteny Database [PMID: 19465509]. Nucleotide and amino acid sequences were compared to the non-redundant sequence databases present at the NCBI (National Center for Biotechnology Information) using the Basic Local Alignment Search Tool (BLAST) [PMID: 2231712]. Multiple sequence alignment was performed using ClustalW algorithm [PMID: 7984417]. Phylogenetic analysis was carried out with the Phylogeny.fr web service [PMID: 18424797] as follows: sequences were aligned with T-Coffe, ambiguous regions (*i.e*. containing gaps and/or poorly aligned) were removed with Gblocks, the phylogenetic tree was reconstructed using the maximum likelihood method implemented in the PhyML program and tree rendering obtained with TreeDyn. The GenBank accession numbers of the amino acid sequences used for multiple sequences alignment and phylogenetic analysis are listed in Table ST1 (supplementary information). Protein subcellular localization prediction was performed using TargetP [PMID: 10891285] and Wolf PSORT [PMID: 17517783] algorithms.

### 2.2 Fish breeding, embryo collection and treatments

Wild type zebrafish AB strain or the transgenic lines Tg(*− 2.4 Kb neurod*:EGFP), hereinafter named Tg(*neurod*:EGFP) (Casari et al., 2014), Tg(*− 2.4 Kb neurod*:EGFP-*sox10*:dsRed) abbreviated as Tg(*neurod*:EGFP-*sox10*:dsRed) (Kucenas et al., 2008), Tg(*gata1a*:dsRed) (Traver et al., 2003) and Tg(*gata1a*:dsRed-*fli1a*:EGFP) (Lawson and Weinstein, 2002) were used for the experiments. They were all kept in tanks containing 3–5 l of water at 28 °C on 14 h light/10 h dark cycle (Westerfield et al., 1990). Adult zebrafish were bred by natural crosses and collected embryos were staged according to Kimmel et al. (1995). Embryos were raised at 28 °C in fish water (0.1 g/l Instant Ocean Sea Salts, 0.1 g/l sodium bicarbonate, 0.19 g/l calcium sulfate, 0.2 mg/l methylen blue, H_2_O) until the desired developmental stage was reached. To examine post-gastrulation stages, regular fish water was replaced by 0.0045% PTU (1-phenil-2-thiourea, Sigma) solution. The embryos were dechorionated by hand using sharpened forceps and then fixed in 4% (wt/vol) paraformaldehyde 1 × PBS overnight at 4 °C (or 2 h at room temperature), into 2-ml tubes, dehydrated through sequential washes in 25%, 50%, 75% methanol/PBS, 100% methanol and stored at least overnight at − 20 °C. The oldest age at which the zebrafish embryos were sacrificed is 96 hours post fertilization (hpf). Euthanasia of embryos has been carried out by prolonged immersion in tricaine methane sulfonate (400 mg/l). To ensure death, bleach solution (6.15% sodium hypochlorite) was added at 1 part bleach to 5 parts water.

To determine the effects of pantethine, CoA and vitamin B5 (Sigma) upon embryos development, the specific drug was added directly in the fish-water 2 or 5 hpf (blastula or gastrula developmental stage); dose-curve experiments were performed with concentrations ranging from 20 to 500 μM and the morphology observed at 24 and 48 hpf. Heart beat rate was determined by direct counting in at least 10 different embryos from 6 microinjection experiments.

Although current Italian rules (Art. 7 D.L. 116/92 and Art. 8 22/04/1994) do not require a formal approval for biomedical research on zebrafish embryos, a project entitled “Utilizzo dell'embrione del pesce teleosteo *D. rerio* – zebrafish – per lo studio di patologie umane” (Use of the teleost fish *D. rerio* – zebrafish – for the study of human disease) has been presented on 8/10/2010 and approved by the Ministero del Lavoro, della Salute e delle Politiche Sociali (Ministry of Labour, Health and Social Policy). The approval has been renewed after a new request submitted on 10/10/2013. The Zebrafish Centre in Padova is working under the approval Prot. 12489, 20/02/2013, from the University of Padova Ethics Committee for Animal Experimentation (Project 74 BIS/2012: “Use of Zebrafish – *D. rerio* – as a model for vertebrate development and human pathology”). On the whole, animal experiments were carried out according to the EU Directive 2010/63/EU.

### 2.3 RNA extraction and real time PCR

Total RNA was extracted from 30 embryos for each different developmental stage analyzed, frozen in liquid nitrogen, using TRI-Reagent (Sigma) according to manufacturer's protocol. For tissues dissection, the adult fishes were killed by an excess of ethyl 3-aminobenzoate methanesulfonate salt solution (Sigma). RNA was quantified using the My Spect spectrophotometer (Biomed) and controlled by electrophoretic separation on a 1% TAE-agarose gel. 1.0 μg of total RNA was retro-transcribed to cDNA using Im-Prom reverse transcriptase (Promega) and oligo(dT) primers following the manufacturer's protocol. Primers were designed by the PrimerQuest and Real Time PCR Tool from IDT (see Table ST2 in supplementary information). Real-Time PCR was performed using the Eco-Illumina system. Reactions were performed in a 10 μl volume, with 100 μM of primer P1 and P2 for *pank2* and A1 and A2 for *actb1*, 12.5 μl of Syber Green Master Mix (Biorad) and 20 ng of cDNA. The amplification profile consisted of a denaturation program (95 °C for 1 min), 40 cycles of two steps amplification (95 °C for 15 s and 60 °C for 30 s) followed by a melting cycle. Each reaction was performed in triplicate and when possible *actin beta 1* was used as a reference gene. Relative levels of expression were calculated by the ΔΔCT method and with respect to the 2-cell stage.

### 2.4 *pank2* cDNA isolation and expression in mammalian cells

The full length *pank2* cDNA was amplified by RT-PCR from the RNA obtained from the 2-cell stage with Pfu DNA polymerase (Promega) and the primers P3 and P4 (Table ST2). The P3 primer was designed to add the FLAG-tag sequence at the 3′ of *pank2* cDNA. The amplified product was digested with *NheI* e *BamHI* and subcloned in the pcDNA3.1 vector digested with the same restriction enzymes. Automated DNA sequencing of the recombinant construct confirmed the sequence of the cloned insert. Cos7 and HeLa cells were cultured in DMEM (PAA Laboratories) supplemented with 10% fetal bovine serum (Euroclone), 1 mM l-glutamine and 40 μg/ml gentamicin. SH-SY5Y cells were cultured in Minimal Essential Medium/Ham's F-12 (PAA Laboratories) with 10% fetal bovine serum, 40 μg/ml gentamicin and 1 mM l-glutamine. For transfection, 10^5^ cells were seeded in chambers mounted on glass slides and after 24 h exposed to 1.0 μg of pcDNA-*pank2*-FLAG cDNA and 3.0 μl of ViaFect reagent (Promega) according to manufacturer's instructions. 24–48 h after transfection the cells were eventually incubated with Mitotracker (Life Technologies) 25 nM, for 10 min in DMEM without FBS and then washed three times with PBS, fixed in 4% paraformaldehyde in PBS for 10 min at room temperature and permeabilized with PBS/0.1% Triton-X100 at 4 °C for 15 min. Cells were incubated for 30 min in wash medium (PBS/5% BSA) and then with anti-FLAG primary antibody (1:300, Sigma) diluted in PBS/1% BSA for 16 h. After three washes in wash medium they were incubated with anti-mouse secondary antibody conjugated to the fluorescent dye AlexaFluor 488 (1:300, Life Technologies) for 1 h, washed three times in wash medium and once with PBS alone. Coverslips were then mounted with aqueous mounting medium (Sigma) and analyzed at the microscope.

### 2.5 Riboprobes synthesis and whole-mount *in situ* hybridization

To synthesize the riboprobes for the detection of zebrafish *pank2* transcripts, we amplified specific regions by PCR using as template the cDNA from the 2-cell stage and oligonucleotide primers P5-P6 and P7-P8. The amplification conditions were the following: 2 min at 95 °C, 30 cycles at 94 °C for 30 s, 60 °C for 30 s, 72 °C for 1 min, followed by final extension at 72 °C for 5 min. The PCR products were subcloned with the pGEM-T-Easy system (Promega), and verified for sequence and orientation of the inserts. Antisense and sense RNA probes were obtained by *in vitro* transcription of the cloned cDNAs with T7 or SP6 RNA polymerase, using a digoxigenin labeling mixture according to manufacturer's instructions (Roche). Whole-mount *in situ* hybridization (WISH) was performed according to a standard method (Thisse and Thisse, 2008). Briefly, embryos and larvae were collected, dechorionated and incubated at 28 °C at different stages. Embryos were fixed overnight in 4% paraformaldehyde (PFA) at 4 °C, dehydrated through an ascending methanol series and stored at − 20 °C. After treatment with proteinase K (10 μg/ml, Roche), the embryos were hybridized overnight at 68 °C with DIG-labeled antisense or sense RNA probes (400 ng). Embryos were washed with ascending scale of Hybe Wash/PBS and SSC/PBS, then incubated with anti-DIG antibody conjugated with alkaline phospatase over night at 4 °C. The staining was performed with NBT/BCIP (blue staining solution, Roche) alkaline phosphatase substrates. When different type of probes (sense *vs* antiense) or fish (injected *vs* not-injected) had to be compared, all incubations were carried out at the same time, at the same probe concentration and, when possible, with the same reagents and solutions. WISH images were taken with a Leica MZ16F stereomicroscope equipped with DFC 480 digital camera and LAS Leica Imaging software (Leica). Magnification: 50 ×, 63 ×, and 80 ×.

### 2.6 Staining with acridine orange and anti-phosphohistone H3 antibody

Acridine orange (AO) staining is a nucleic acid selective metachromatic stain technique that can identify cell death (Perkins et al., 2005). For AO staining, embryos at 2 dpf were *in vivo* dechorionated and incubated for 30 min in fish water containing acridine orange at 10 mg/l. Embryos were then rinsed three times in fish water, anesthetized with tricaine, positioned in 2% methyl cellulose and quickly imaged under a dissecting microscope with green fluorescent filter. For cell proliferation analysis, we exploited the mitosis-specific marker phosphohistone H3 (PHH3) (Hendzel et al., 1997). After PFA-fixation and methanol storage, embryos were rehydrated and incubated two times in PBST (PBS/1% Triton X-100), one time in distilled water and 7 min in acetone at − 20 °C. After one wash in distilled water and one wash in PBST, embryos were pre-incubated in PBDT (PBS + 1% BSA + 1% DMSO + 0.5% Triton X-100) plus 2% goat serum (Sigma G9023), and then incubated over night at 4 C in PBDT/goat serum containing a rabbit anti-PPH3 antibody (1:1000, Merck Millipore 06-570). After four washes, 15 min each, in PBDT, embryos were incubated over night at 4 °C in PBDT containing a goat anti-rabbit AP-conjugated antibody (1:1000, Merck Millipore 12-448). After four washes, 15 min each, in PBDT, embryos were stained by NBT/BCIP protocol, post-fixed in PFA and mounted in 87% glycerol/PBS for documentation.

### 2.7 Microinjections

To knock-down the expression of Pank2 protein, the P2-MO splice-blocking morpholino (GeneTools, LCC) was synthesized targeting the exon1-intron1 boundary (Table ST2). Different amounts of morpholino were initially injected into wild type embryos (Fig. S4), to determine the optimal concentration of 1 pmol/embryo as the one appropriate for these experiments and with no toxic effects. A standard control morpholino oligonucleotide (ST-MO) was used as negative control (Table ST2). The morpholinos were injected in 1 × Danieau buffer (pH 7.6) into 1-to 2-cell stage embryos and the dye tracer rhodamine dextran was also co-injected as previously reported (Nasevicius and Ekker, 2000). After microinjection, embryos were incubated in fish water supplemented with 0.003% PTU at 28 °C to prevent pigmentation processes. Embryo development was evaluated at 24 hpf, 48 hpf, and 72 hpf. RT-PCR experiments were performed on RNA extracted from 24 hpf and 48 hpf P2-MO- or ST-MO-injected and wild type embryos, with P9 and P10 primers to demonstrate the absence of a functional gene transcript in morphants. Control RT-PCR amplification on the same RNA was carried out with *eef1a1l1* primers (EF1 and EF2, Table ST2).

### 2.8 Production of *pank2* synthetic mRNA for morpholino phenotype rescue

The coding region of the *pank2* gene was amplified by PCR using P9 and P10 primers (ST1) with Pfu DNA polymerase (Promega). The PCR product was digested with *BamHI* e *XbaI* and cloned in the pCS2 + vector digested with the same restriction enzymes. The plasmid was checked for the sequence and then digested with *PvuII* restriction enzyme; the insert containing the *pank2* cDNA was gel-purified and transcribed with SP6 RNA polymerase using the mMESSAGE mMACHINE SP6 *in vitro* transcription kit (Ambion) according to the manufacturer's instructions. Dose–response curve experiments were performed in wild type embryos to identify the maximum amount of *pank2* mRNA that does not induce phenotypic alterations. The rescue of the morphant phenotype was obtained by co-injecting 1 pmol/embryo of P2-MO together with 50 pg/embryo of synthetic *pank2*-mRNA. The presence of the synthetic mRNA was also confirmed by an RT-PCR performed on injected embryos at 24 hpf with *pank2* (P11 and P12, Table ST2) and *eef1a1l1* specific primers.

### 2.9 Microscopy and confocal imaging

For transverse sectioning, *post in situ* embryos were embedded in gelatin/BSA medium (0.4% Type A porcine skin gelatin, 27% BSA, 18% sucrose in 1 × PBS), plus 1.75% glutaraldehyde. The gelatin blocks were sectioned (30 μm) in PBS using a Leica VT1000S vibratome. The slice were mounted in ImmunoHistoMount (Sigma) and imaged with a compound Leica DMR microscope, equipped with Nomarski optics and a DC500 Leica digital camera. Fluorescence of transgenic lines was visualized using a Leica M165FC dissecting microscope and then a Nikon C2 H600L confocal microscope. For *in vivo* analyses, embryos and larvae were anesthetized with tricaine 0.16 mg/ml and mounted in 1% low melting agarose gel. EGFP and Red fluorescence was visualized by using 488 and 561 nm lasers, respectively, through 20 × and 40 × immersion objectives (Nikon). All images were analyzed with Nikon and Volocity 6.0 (Perkin Elmer) software.

### 2.10 Statistical analysis

The data are reported as the mean +/− SD values or as representative of at least three independent experiments with similar results. Statistically significant differences between different types of embryos were calculated by one-way ANOVA analyses; *, ** indicated pb < 0.05, pb < 0.01 respectively.

## 3 Results

### 3.1 Identification and characterization of zebrafish *pank2* gene

**Fig. S1:**
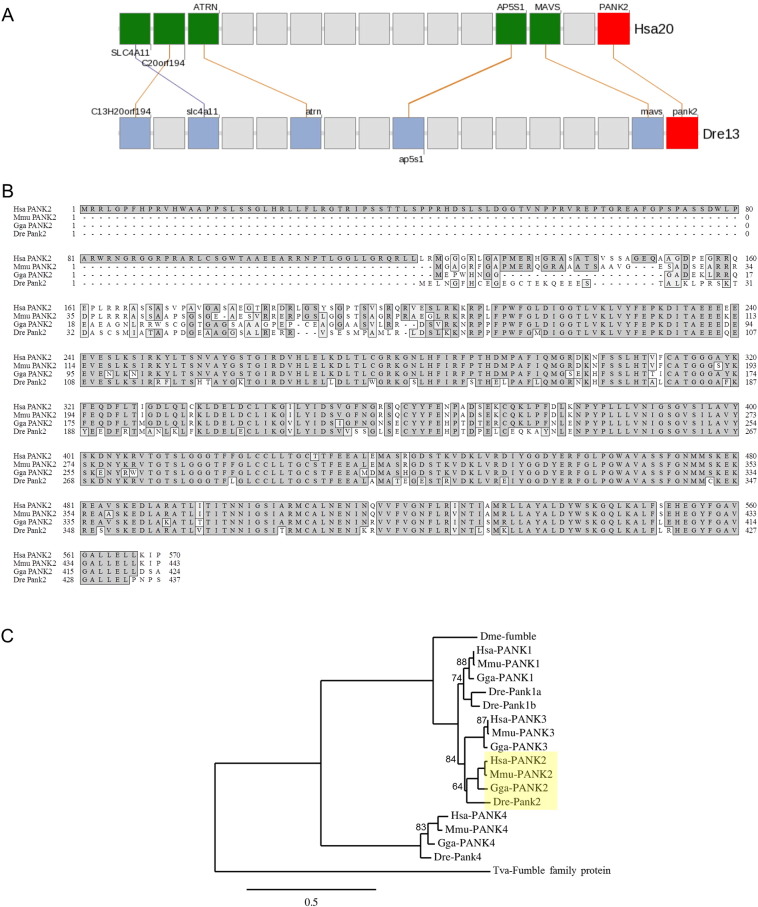
Bioinformatic analysis of zebrafish *pank2* gene and protein sequences. (A) Gene trace synteny analysis around the *PANK2* locus between *Homo sapiens* chromosome 20 (Hsa20) and *Danio rerio* chromosomes 13 (Dre13) generated using the Synteny Database (50-gene sliding window). Genes are drawn as squares, with their order preserved. Colored squares are members of the cluster while gray squares represent genes in the interval but that do not have orthologs or paralogs in the other segment. *PANK2* genes are drawn as red squares. Lines connecting squares between the two clusters represent putative orthologous gene pairs. (B) Multiple sequences alignment of human (Hsa) mouse (Mmu), chicken (Gga) and zebrafish (Dre) PANK2 proteins obtained using the ClustalW2 software. Residues that are identical are shown on a dark gray background; those on a light gray background are the conservative substitutions. (C) Rooted tree showing phylogenetic analysis results for human (Hsa), mouse (Mmu), chicken (Gga) and zebrafish (Dre) pantothenate kinase family of proteins, as well as the fumble polypeptides from *Drosophila melanogaster* (Dme) and *Tricomonas vaginalis* (Tva). The tree clade including PANK2 polypeptides is highlighted in yellow. The horizontal bar represents a distance of 0.5 substitutions per site. Branch support values are displayed in percentage (support values > 90% are not shown). The tree, generated using the Phylogeny.fr web service, was rooted using the *Tricomonas vaginalis* Fumble family protein as an outgroup.

The Gene and HomoloGene databases at NCBI indicate a single zebrafish ortholog of the human *PANK2* gene. *D. rerio pank2* (Gene ID: 570866) is also present in the ZFIN database with the following ID: ZDB-GENE-070112-1952. It is located on chromosome 13 and similarly to the human counterpart, is organized in seven exons and harbors the *miR-103* gene within the fifth intron (data not shown). The analysis of the genomic region surrounding *pank2*, carried out using the Synteny Database, allowed to identify conserved synteny between human chromosome 20 and *D. rerio* chromosome 13 (Supplementary information, Fig. S1A). Similar results have been obtained using the Genomicus synteny browser (data not shown). *pank2* encodes a protein of 437 aa (RefSeq NP_001074075.2) that is highly conserved in vertebrates, and homologous sequences can be found in lower eukaryotic and prokaryotic organisms. A multiple sequence alignment among PANK2 polypeptides from man, mouse, chicken and zebrafish (Fig. S1B) indicates a high level of identity among the four polypeptides, with the exception of the first 124 amino acids at the N-terminus of the human sequence, here represented by the mitochondrial isoform 1 preproprotein (NP_705902). In a local alignment analysis zebrafish Pank2 shows 72% and 77% identity to the human and mouse polypeptides, respectively. A phylogenetic analysis, carried out including all the pantothenic acid kinases identified in the four vertebrates considered for the multiple sequence alignment, shows that the *D. rerio* protein under investigation correctly segregates in the PANK2 clade (Fig. S1C). RNA-Seq data from the Wellcome Trust Sanger Institute Zebrafish Transcriptome Sequencing Project indicate that *pank2* is transcribed throughout development and in adult fish (data not shown) [PMID: 22798491]. The presence of RNA-Seq reads from 2-cell stage embyos indicates that the gene is maternally expressed. Data inferred from Expression Sequence Tag (EST) profile provide evidence that the gene is particularly expressed in the brain of adult fish.

Zebrafish *pank2* ORF was subcloned in pcDNA3.1 vector with a 3′ sequence extension coding for the FLAG epitope tag. Cos7 and HeLa cells were transfected with the plasmid and the distribution of the flag-tagged protein analyzed by immunofluorescence. In transfected cells we detected a diffuse staining, presumably cytosolic often associated with a nuclear signal, as evidenced by DAPI co-staining (Fig. S2). We did not find any evident overlap with the mitochondrial marker Mitotracker.

### 3.2 Developmental expression of zebrafish *pank2* gene

**Fig. 1:**
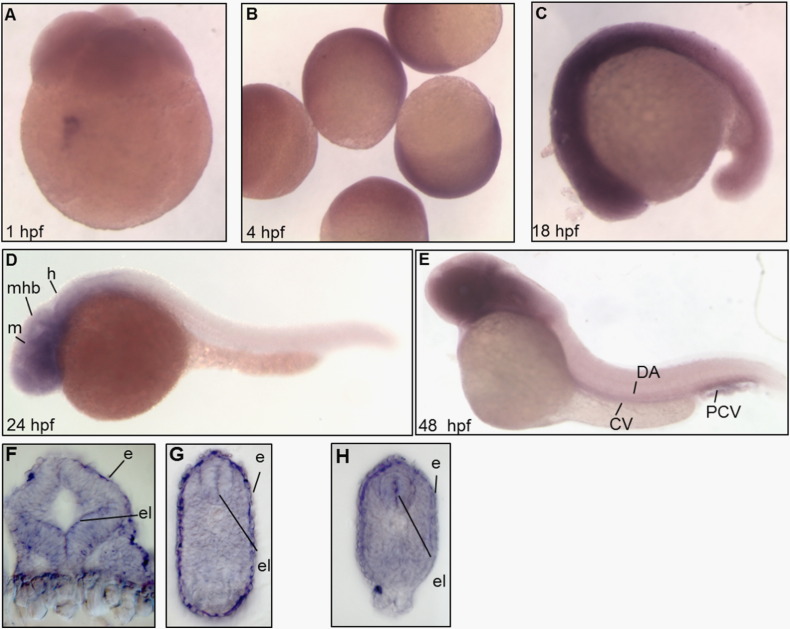
Developmental expression of zebrafish *pank2*. WISH was performed from 1 to 48 hpf using a *pank2-*specific, DIG-labeled antisense probe of 625 bp. From the “cleavage period” to the somitogenesis the *pank2* signal is diffuse (A, B, C). At 24 hpf the transcript is present in defined CNS regions (midbrain, hindbrain and midbrain–hindbrain boundary). At 48 hpf the signal in the brain region is still present and also appears in the main vessels and in vessels of the vascular plexus in the tail region (E). A, B, C dorsal view, D and E lateral view, anterior to the left. Cross-sections at the level of the head and trunk of 24 (F, G) and 48 hpf embryos (H). Abbreviations: m, midbrain; mhb, midbrain–hindbrain boundary; h, hindbrain; da, dorsal aorta: cv; cardinal vein; pcv, post cardinal vein; e, epidermis; el, ependymal layer. The figure shows representative images from at least three different experiments, with 20 embryos/stage.

To study *pank2* gene expression and distribution during zebrafish embryonic development, we carried out WISH experiments at different developmental stages: 4-cell, 30% epiboly, 18-somite stage, 24 and 48 hpf. Two different regions of the *pank2* mRNA were amplified by RT-PCR, cloned in pGEM-T vector and the sequence was confirmed. The plasmids were then used to synthesize the specific digoxigenin-labeled antisense and sense riboprobes by *in vitro* transcription with T7 or SP6 RNA polymerase. To assess the specificity of hybridizations, sense probes were used in parallel and with the same experimental conditions; no staining was detected in any embryo at all stages (Fig. S3A, B). The WISH performed with two different antisense riboprobes revealed that *pank2* is a maternal gene, expressed in zygotes and at early stages of development (epiboly) (Fig. 1A–C). During somitogenesis a more defined expression pattern appeared in the rostral part of the embryo at the level of the developing central nervous system (CNS). At 24 hpf *pank2* is transcribed in defined structures such as forebrain, midbrain and cerebellum (Fig. 1D); at 48 hpf *pank2* continues to be mainly expressed in the central nervous system but it also appears in vascular structures such as the axial vein, the dorsal aorta and the caudal plexus (Fig. 1E). Hystological sections of embryos hybridized with *pank2* riboprobe were performed at 24 and 48 hpf, revealing intense signals localized in the ependymal layer of the CNS and in the epidermis. In parallel, we analyzed *pank2* expression by real time qRT-PCR performed with 20 ng of cDNA from different developmental stages (Fig. S3C) and adult tissues (Fig. S3D). The analysis shows highest levels of the transcript at the 1/2-cell stage, confirming that the *pank2* gene is maternally expressed. *pank2* mRNA partially drops at lower levels in the initial hours of development and raises back to higher values at 48 and 72 hpf. In adult animals, the *pank2* transcript is expressed at the highest level in the brain and in the eyes. Overall the analysis suggests a role for *pank2* in CNS early differentiation and function and possibly also in vasculogenesis and/or angiogenesis.

### 3.3 Morphological investigation of the phenotype induced by *pank2* down-regulation

**Fig. 2:**
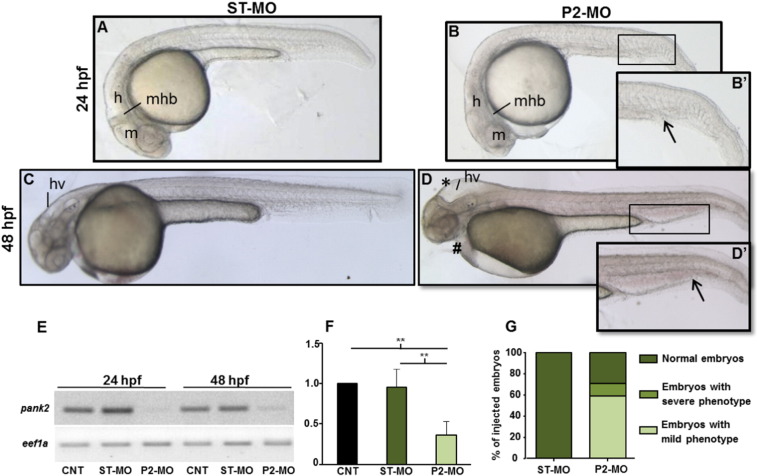
Effects of *pank2* morpholino during zebrafish development. Standard (ST-MO) and *pank2* morpholino (P2-MO) were micro-injected at 1/2-cell stage (1 pmol/embryo) and the phenotype observed at 24 and 48 hpf. At both developmental stages, morphants showed abnormalities in the brain region, presence of hydrocephalus (*) and heart edema (≠) (D), severe perturbation of main vessels (dorsal aorta and caudal vein) and vascular plexus in the tail (arrowhead and magnification B’, D’). E) Semi-quantitative RT-PCR analysis of *pank2* transcript levels in non-injected (CNT), ST-MO- and P2-MO-injected embryos at 24 and 48 hpf. F) Densitometric analysis of RT-PCR products resolved by agarose-gel electrophoresis. * = P < 0.001. G) Quantitative analysis of the phenotype observed in P2-MO-injected embryos at 48 hpf. Two different phenotypes could be distinguished: a severe one (12%) with head malformations, presence of hydrocephalus and evident edema in the caudal plexus, severe defects in anterior-posterior axis and delay in development; a milder phenotype (59%) with not well-defined brain areas, less severe hydrocephalus and edema in caudal plexus. The graph shows the analysis performed in six independent experiments on a total of 562 injected embryos. Abbreviations: hv, hindbrain ventricle, h, hindbrain, m, midbrain, mhb, midbrain–hindbrain boundary.

To investigate the function of *pank2* gene during zebrafish development we designed an antisense morpholino-oligotargeting the exon 1/intron 1 boundary of the gene (P2-MO). A standard morpholino with an unrelated sequence (ST-MO, Gene-Tools) was used as control in all experiments. Both morpholinos were injected at one/two-cell stage and embryos were collected at the indicated developmental stages. Dose curve experiments were performed to set the optimal concentration of 1 pmol/embryo (Fig. S4A). Normally, 85% of P2-MO-injected embryos survived (n = 232/276), compared to 99% of control embryos injected with ST-MO (n = 282/286). The RT-PCR analysis of *pank2* morphants at 24 and 48 hpf confirmed the targeting efficacy of the splice-inhibiting morpholino. A band corresponding to the endogenous *pank2* transcript was clearly detected in non-injected and control-injected embryos, while the expected band was reduced by 65% (P < 0.01) in P2-MO-injected embryos (Fig. 2E, F). To evaluate possible interference of the morpholino upon expression of other *pank* paralogs, we quantified *pank1a* and pank1*b* mRNA levels by RT-PCR at 2-cell and 24 hpf stages in control and P2-MO-injected embryos, without detecting any difference among the analyzed samples (Fig. S4B). Then, we carefully compared the morphology of P2-MO-injected embryos at 24 and 48 hpf with that of non-injected and control ones. A summary of the main phenotypic features is presented in Table 1. At 24 hpf P2-MO-injected embryos showed clear abnormalities in the development of CNS and vascular structures. The brain structures were not well-defined, with a clear reduction of the hindbrain (Fig. 2A, B). A nascent edema was evident in heart region and caudal plexus (Fig. 2B’). The phenotype was more evident at 48 hpf, when a significant percentage (73%, n = 209/283) of embryos showed abnormal head development with smaller eyes and hydrocephalus in the midbrain and hindbrain ventricle. Additionally, we could often observe reduction of the antero-posterior axis and presence of edema in the cardiac region. The heart rate was significantly reduced, with a mean of 120 bpm in control fish *vs* 85 in P2-MO-injected embryos (Fig. S5) and blood circulation appeared to be slower at the visual observation (not shown). The caudal plexus was irregular and hemorrhagic (Fig. 2C, D, D’). Altogether the analysis of embryos from 6 different microinjection of a specific *pank2*-morpholino revealed a clear phenotype, with specific perturbation of central nervous and vascular system, suggesting the requirement for *pank2* expression in normal development of these tissues and organs in zebrafish..

**Fig. 3:**
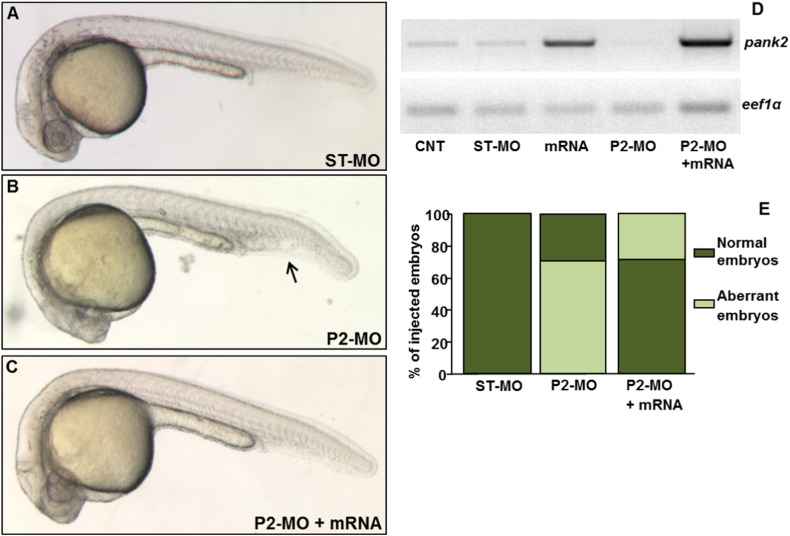
Rescue of the *pank2* knock-down phenotype by co-injection of zebrafish *pank2* mRNA. Lateral views of ST-MO- (A), P2-MO- (B) and P2-MO/*pank2* mRNA-injected embryos (C) at 24 hpf. D) Gel electrophoresis of RT-PCR products for *pank2* and *eef1a1l1* mRNAs performed on described samples. The figure shows a representative experiment out of five with similar results. E) The graph shows the statistical analysis result from the morphological evaluation of embryos.

To validate the cause–effect relationship between lack of *pank2* and the phenotype observed in P2-MO-injected embryos, we performed phenotypic/functional rescue experiments by co-injection of P2-MO and exogenous *pank2-*mRNA. While the single injection of high doses of *pank2* mRNA (500–200 pg/embryo) resulted in significant abnormalities and high mortality (> 90%, not shown), the dose of 50 pg/embryo was not lethal and did not induce significant morphological changes when co-injected with the ST-MO or alone (not shown); on the contrary, co-injection of 50 pg of exogenous mRNA with 1 pmol/embryo of P2-MO was able to restore the wild type phenotype in 71% of injected embryos (n = 108/152, in a total of 5 microinjection) (Fig. 3A–E). The efficacy of the mRNA micro-injection was monitored by a specific RT-PCR, that revealed a significant increase of *pank2* mRNA on injection of exogenous mRNA (Fig. 3D).

**Fig. 4:**
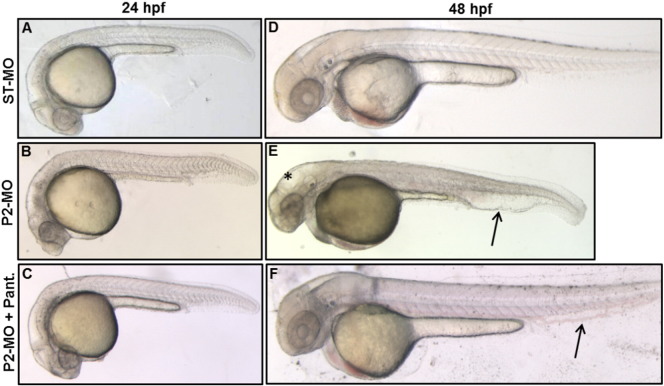
Rescue of the *pank2* knock-down phenotype by pantethine. Morphological comparison of ST-MO- (A, D) and P2-MO-injected embryos (B, E) with P2-MO-injected embryos treated with pantethine 30 μM (C, F) at 24 and 48 hpf. A high percentage of embryos exposed to pantethine showed correction of the aberrant phenotype, with absence of edema in the caudal plexus (arrowheads) and hydrocephalus (asterisk).

PANK2 is a relevant enzyme in CoA biosynthesis and defects in its functioning have been successfully reverted in cellular and animal models by pantethine supplementation in culture medium or in diet (Rana et al., 2010, Brunetti et al., 2014). When added to fish-water, pantethine showed significant toxicity at doses higher than 50 μM (Fig. S6A). Addition of pantethine at 30 μM at 5 hpf (gastrula stage) restored the wild type phenotype in a large percentage (81%) of P2-MO-injected embryos (n = 126/156, out of 3 microinjections) at 24 hpf (Fig. S6B), without affecting efficacy of the injected morpholino (not shown). Most embryos injected with P2-MO and treated with pantethine showed no perturbation of brain morphology and no vascular alteration and signs of edema in the caudal plexus at 24 hpf and 48 hpf, and were very similar to non-injected and ST-MO injected embryos (Fig. 4). On the contrary, 50 μM vitamin B5 did not affect the morphology shown by P2-MO-injected embryos (Fig. S7).

**Fig. 5:**
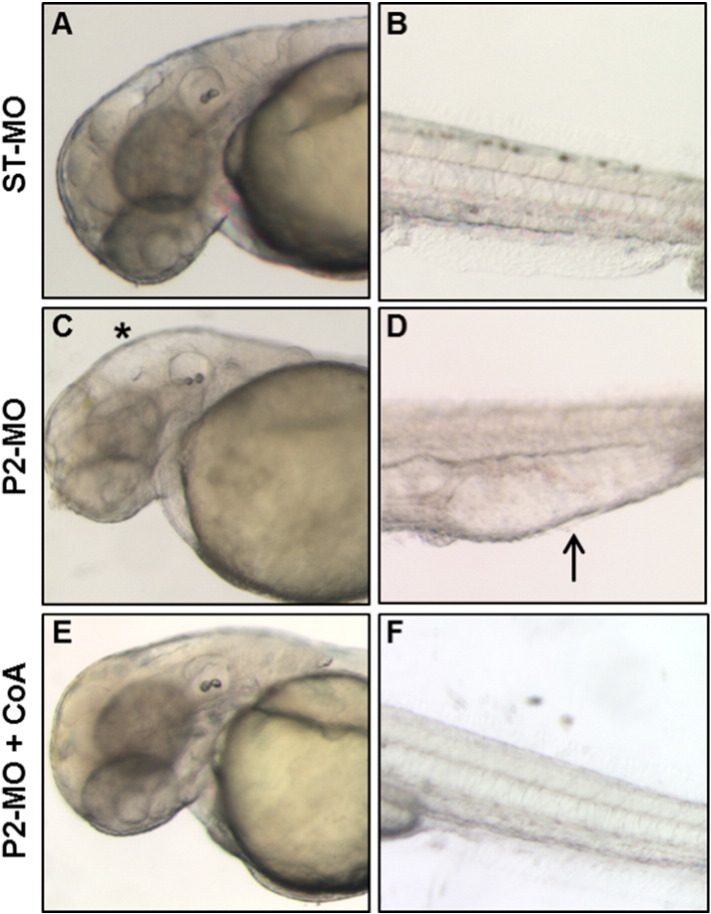
CoA administration allows normal development in P2-MO-injected embryos. Morphological comparison at 48 hpf of ST-MO- (A, B) and P2-MO-injected embryos (C, D) with P2-MO-injected embryos treated with 100 μM CoA (E, F). The magnification of heads (A–E) and tails (B–F) of representative embryos is shown. Addition of CoA to fish-water at blastula stage prevents all morphological alterations observed in embryos upon *pank2* down-regulation.

Addition of CoA to the growth medium of *Drosophila* Schneider's S2 cells depleted of dPANK/Fbl was able to restore normal levels of protein acetylation, apparently by replenishing intracellular CoA stores (Siudeja et al., 2011). Since zebrafish embryos are easily permeable to small molecules and largely used for high-throughput screening of chemical compounds (Tan and Zon, 2011), we decided to directly verify whether CoA itself added to fish-water could prevent the aberrant development due to *pank2* down-regulation. CoA had some toxic effect on zebrafish embryos only at concentrations higher than 500 μM (data not shown). 100 μM CoA was added to the water of embryos two hours after morpholino injection, and the phenotype was observed at 48 hpf. While most embryos exposed only to P2-MO showed the usual phenotype with hydrocephalus and edema of heart and tail, the vast majority (82%) of the embryos (n = 87/105 out of 3 microinjection) also treated with CoA had a normal phenotype, similar to that of ST-MO-injected embryos (Fig. 5). These results suggest that down-regulation of *pank2* can lead to a shortage of CoA in specific cells and tissues in zebrafish embryos, that can be compensated by metabolites entering the CoA biosynthetic pathway downstream of panthotenate phosphorylation (pantethine, CoA). At the same time, they suggest that other *pank* paralogs cannot compensate for *pank2* downregulation in affected tissues.

### 3.4 Molecular characterization of the neural phenotype in P2-MO-injected embryos

**Fig. 6:**
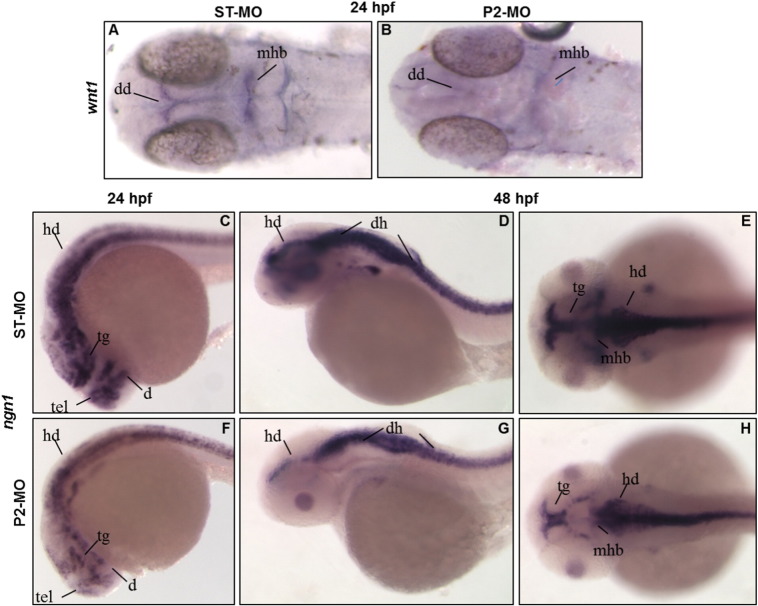
Expression of neural markers is affected in P2-MO-injected embryos. The expression of the neural markers *wnt1* (A, B) and *ngn1* (C–H) was analyzed in ST-MO- and P2-MO-injected embryos by WISH with specific probes. A, B, E, H dorsal views; C–F lateral views. Abbreviations: dd, dorsal diencephalon; tel., telencephalon; tg, tegmentum; mhb, midbrain–hindbrain boundary; hb, hindbrain; dh, dorsal hindbrain.

To gain further insights into the molecular alterations accompanying the CNS phenotype observed after suppression of *pank2*, we investigated the developmental expression pattern of *wnt1* and *neurog1* by WISH. *wnt1* is a factor required for maintenance of expression of several genes in the midbrain–hindbrain boundary (Lekven et al., 2003). Our data showed that about 70% (n = 38/54, 2 microinjections) of *pank2* morphants had a significant down-regulation of *wnt1* expression at 24 hpf (Fig. 6A, B).

Next, we analyzed the expression pattern of *ngn1*, a key transcription factor for zebrafish neurogenesis, and an upstream regulator of *neurod* (Mueller and Wullimann, 2002); it is initially expressed in the neural plate and at later stages it is detectable in tegmentum, dorsal diencephalon, posterior midbrain, midbrain–hindbrain boundary, hindbrain area, optic stalk and spinal cord. At 24 hpf we observed a substantial reduction and partial disorganization of *ngn1* staining in anterior brain areas (including tegmentum, diencephalon and hindbrain) (Fig. 6C, F) in most of P2-MO*-*injected embryos (n = 47/52, two microinjections). Additionally at 48 hpf, a down-regulation in dorsal hindbrain and midbrain–hindbrain boundary was observed in more than 90% of *pank2* morphants when compared to controls (Fig. 6D, E, G, H).

**Fig. 7:**
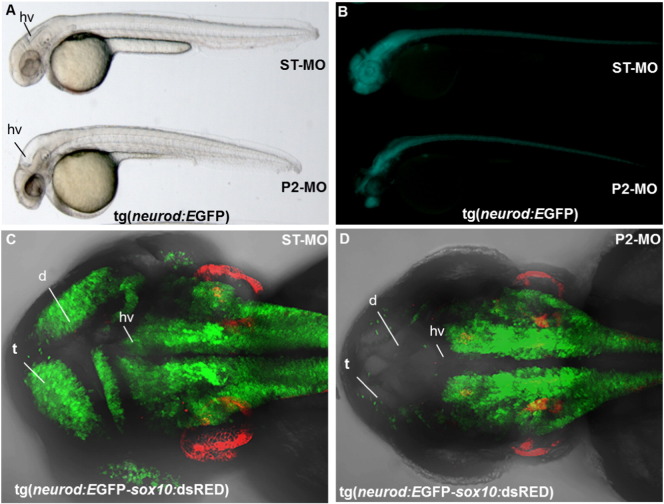
Effects of P2-MO microinjection in transgenic lines Tg(*neurod*:EGFP) and Tg(*neurod*:EGFP*-sox10*:dsRed). Tg(*neurod*:EGFP) transgenic embryos were injected with ST-MO and P2-MO and analyzed at the light (A) and fluorescence microscope (B) at 48 hpf. Confocal analysis of transgenic line Tg(*neurod*:EGFP*-sox10*:dsRed) injected with ST-MO (C) and P2-MO at 48 hpf (D). Abbreviations: t, telencephalon; d, diencephalon, hv, hindbrain ventricle.

**Fig. 8:**
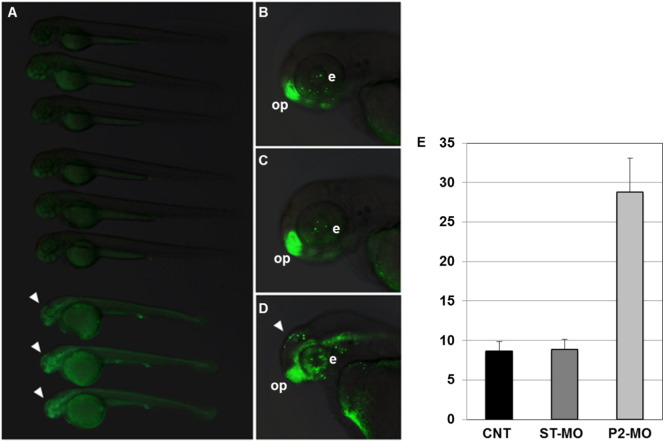
*pank2* knock-down is associated with increased cell death. AO staining of not injected- (B), ST-MO- (C) and P2-MO-injected (D) embryos (all displayed in full size in A) shows an increased number of positive (dying) cells (white arrowheads) in *pank2* morphants, compared to controls. Other positive AO domains, such as eye (e) and olfactory pits (op), are expected at this developmental stage. All embryos are at 48 hpf, in lateral view, anterior to the left. Experiments were performed in duplicate, with at least 30 embryos per condition. The graph in E shows the AO cell counting in the forebrain region of 5 embryos from each condition.

Then, we treated transgenic fluorescent-reporter lines Tg (*neurod*:EGFP) and Tg (*neurod*:EGFP-*sox10*:dsRed) with either ST-MO or P2-MO. *neurod*, as *neurog1*, is a key transcription factor for zebrafish neurogenesis (Mueller and Wullimann, 2002). At 24 hpf it is expressed in telencephalon, diencephalon, symmetric primordial of trigeminal ganglia, olfactory placode and caudally in spinal cord. The *sox10*:dsRED transgene has been included in the analysis as internal control, in order to simultaneously monitor cranial neural crest derivatives (Kague et al., 2012). *In vivo* morphological analysis revealed in P2-MO*-*injected transgenic embryos the same alterations observed in wild type embryos with disorganization of CNS structures, presence of hydrocephalus in midbrain and hindbrain ventricle and smaller otic vesicles (Fig. 7A). At 48 hpf, in the vast majority of *pank2* morphants (n = 37/51) *neurod* expression was severely diminished when compared to control embryos (Fig. 8B). This was confirmed by higher magnification analyses performed on transgenic line Tg(*neurod*:EGFP-*sox10*:dsRed) injected with P2-MO (n = 35/48), where the number of *neurod*-positive cells in telencephalon and dorsal diencephalon was strongly reduced and the hindbrain ventricle was not well defined. On the contrary, we did not observe significant variation in *sox10* expression. (Fig. 7C, D). Taken together the results further confirm the essential role for *pank2* for anterior CNS normal development in zebrafish.

The altered phenotype observed in P2-MO-injected embryos was associated with a significant increase in number of cell death events as assessed by AO staining (Fig. 8). The presence of dead cells was particularly evident in the forebrain but in general it seemed to overlap with the distribution of morphological alterations in morphants. Zebrafish embryos were then stained with an antibody recognizing the phosphorylated form of histone H3 (PHH3), a marker for mitotic nuclei. No significant differences were observed among non-injected-, ST-MO- and P2-MO-injected embryos (Fig. S8) as confirmed by direct counting of positive nuclei performed in the same region analyzed for quantification of cell death events. Overall, these data suggest that the neural phenotype observed in *pank2* deficient embryos is mainly associated to an increased rate of cell death in the forebrain region rather than a decrease of cell proliferation in the same anatomical area.

### 3.5 Injection of P2-MO causes defects in vascular integrity

**Fig. 9:**
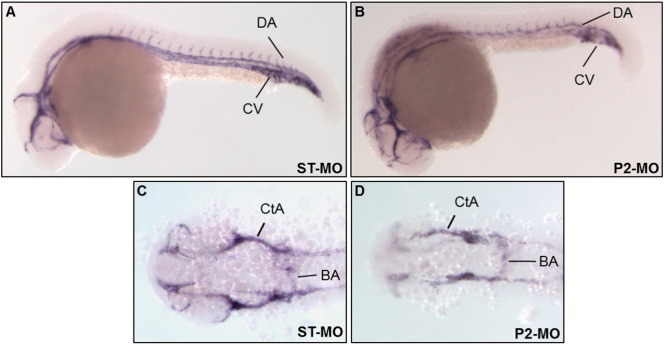
*pank2* down-regulation alters expression of vascular endothelial *cadherin 5*. *Cadherin 5* expression was analyzed by in *situ* hybridization in control embryos (A, C) and *pank2* morphants (B, D) at 24 hpf. Lateral (A, B) and dorsal views (C, D). Abbreviations: DA, dorsal aorta; CV, cardinal vein; CtA, central arteries; BA, basilar aorta.

A more in-depth analysis of the consequences of *pank2* suppression upon development of the vascular system was performed by *in situ* hybridization experiments using a *cadherin 5* specific probe (Montero-Balaguer et al., 2009). This vascular endothelial cadherin is a key protein in early stages of vascular development, being involved in maintenance of inter-cellular junctions in endothelial cells, important structures for control of vascular permeability to cells and plasma proteins and also for correct architecture of vascular network. Embryos were injected with 1 pmol/embryo of P2-MO and analyzed at 24 hpf. At these developmental stages, about 75% (n = 39/52, out of 2 microinjections) of the injected embryos showed a significant down-regulation of the *cadherin 5* signal in main vessels (axial vein and dorsal aorta) and in intersomitic branches sprouting from them (Fig. 9).

**Fig. 10:**
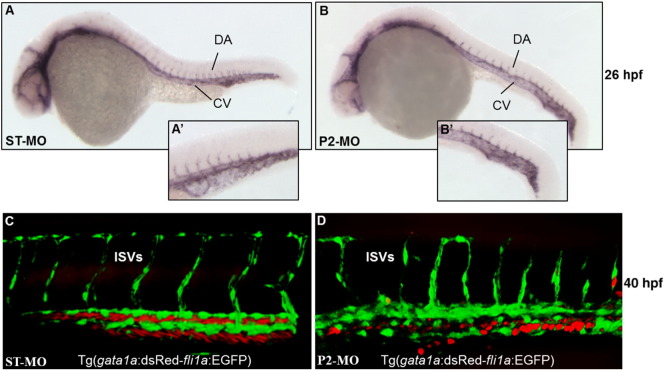
*pank2* knock-down affects trunk vessel integrity. WISH analysis of *fli1a* expression at 26 hpf in ST-MO- and P2-MO-injected embryos (A, A’, B, B’). We injected 1 pmol/embryo of ST-MO (C) and P2-MO (D) in Tg(*gata1a*:dsRed*-fli1a*:EGFP) embryos and analyzed the phenotype by confocal microscopy at 40 hpf. Abbreviations: DA, dorsal aorta; CV, caudal vein; ISVS, intersegmental vessels..

Very similar results were obtained using another endothelial-vascular marker: *fli1a.* At 26 hpf *pank2* morphants hybridized with the *fli1a* riboprobe showed a significant alteration in the formation of intersegmental vessels (Fig. 10A, A’, B, and B’). We injected the P2-MO in the double transgenic fish line Tg(*gata1a*:dsRed-*fli1a*:EGFP) that expresses dsRed and EGFP proteins under *gata1a* and *fli1a* promoter, respectively. Gata1 is a zinc-fingered transcription factor necessary for terminal differentiation of erythroid, eosinophilic and mast cell lines (Cantor, 2005); hence it is a tool for detecting organs responsible for blood cell production and erythrocytes (Long et al., 1997); Fli1a is a crucial transcription factor for vascular development (Liu et al., 2008), being expressed in the endothelial precursors, which develop into cranial, axial and intersegmental blood vessels. We analyzed injected embryos at 48 hpf by confocal microscopy. In the vast majority of P2-MO-injected embryos (n = 52/73, 71%, out of 2 microinjections) we observed a partial disorganization of intersegmental vessels and malformation of the aorta and cardinal vein. A significant delay in establishment of anastomosis between contiguous intersegmental vessels was evident; several vessels lacked connection with the rest of vasculature and presented altered lumen and signs of initial regression (Fig. 10C, D). Blood flow is important in formation of stable vessels that integrate in a functional network and seems to be required in certain vessels (ISVs) for opening of vascular lumen. In P2-MO*-*injected embryos we observed an altered distribution of red cells, suggesting a significant disorganization of endothelial cells and dysmorphic vascular lumen (Fig. 10C, D). The difference in shape of red blood cells (dsRed positive) in control fish (rod-like) and morphants (round) can be attributed to the drastic reduction of blood flow velocity in P2-MO-injected fish. The results were confirmed by confocal microscope analysis at 80 hpf of transgenic fish Tg(*gata1a*:dsRed) and Tg(*fli1a*:EGFP) where we also observed the presence of hemorrhages in head and heart regions of injected embryos, probably due to loss of vessel integrity and maintenance (Fig. S9). Our data suggest that *pank2* could play a critical role in junctional complex maturation, an important event for permeability modulation and for correct formation of the vascular network..

## 4 Discussion

Model organisms provide an important platform for analysis of human disease processes and development of therapies. The zebrafish, with a vertebrate biology, easy methods of genetic manipulation, and specific brain regions that are conserved and comparable to human counterparts, appears as a good model to study the pathophysiology of human neurodegenerative disorders. In particular, the possibility to investigate embryonic development may provide relevant insight into the pathogenesis of disorders with very early onset, as many of those included in the NBIA category. In this work, we provided the first *in vivo* functional characterization of *pank2*, the zebrafish ortholog of human *PANK2*, the gene mutated in PKAN, and documented its essential role during vertebrate development. Zebrafish Pank2 protein shows high sequence identity with mammalian counterparts but lacks the stretch of about 120 amino acids present at the N-terminus of the human sequence, that was shown to contain the mitochondrial targeting signal (Alfonso-Pecchio et al., 2012). Indeed, when expressed in mammalian cell lines zebrafish *pank2* localizes in cytosol and nucleus. Due to the lack of a zebrafish specific antibody, we could not verify whether this cellular localization represents the physiological condition in the fish. *In silico* analysis of human, mouse, and zebrafish amino acid sequences performed with TargetP and Wolf PSORT indicate a clear mitochondrial localization exclusively for the human protein. Interestingly, also the mouse protein lacks the N-terminal sequence of the human mitochondrial isoform, but according to some studies (Johnson et al., 2004, Brunetti et al., 2012), it localizes in the mitochondria. If confirmed, the difference in cellular distribution could be an indication of selective properties characterizing the human enzyme. Nonetheless, human and zebrafish genes share the important and conserved feature of being expressed at high levels in brain and particularly in neurons, as shown by qRT-PCR and WISH analysis, which makes the zebrafish model a relevant system to investigate *pank2* function and developmental role, with potential outcomes for comprehension of PKAN pathogenesis. Interestingly enough, the *in situ* hybridization decorated also cells of the ependymal layer and gave an intense, specific signal also in main axial vascular structures (dorsal aorta and cardinal vein), findings that could indicate novel tissues and pathways potentially affected by Pank2 defects. The gene shows a clear maternal contribution at early developmental stages; indeed, it is already present at lower levels at 256-cell stage and it is then maintained up to 24 hpf. Later on, it raises to higher levels at 48 and 72 hpf. The loss-of-function studies, using the morpholino approach, confirmed WISH data and evidenced a relevant role for *pank2* in development of CNS and vascular structures. Significant edema in the midbrain/hindbrain ventricle and a severe perturbation of brain morphology appeared at 24 hpf and worsened at 48 hpf. This was associated with a clear loss of neural cells particularly in telencephalon and diencephalon as shown by WISH with *wnt1* and *neurog1* neural marker riboprobes and by the fluorescent pattern in Tg(*neurod*:*EGFP-sox10*:*dsRed*) line exposed to P2-MO. Our cell death and proliferation assays point to a major role for cell loss, compared to decreased mitosis, in the determination of the observed phenotypes. This could indicate that *pank2* function is required for normal development and maintenance of neurons in these brain areas and is of potential interest for PKAN pathology, that specifically affects the globus pallidum. In adult fish, telencephalon, and particularly the ventral zone contains brain nuclei proposed to be homologous to vertebrate subpallium, including the pallidum (Northcutt, 1981, Northcutt, 1995, Ganz et al., 2012). Since *neurod* is mostly expressed in dorsal telencephalon, we cannot infer from our data the presence and role of *pank2* in pallidal neurons; application of other more specific neural markers such as *nkx2.1b*, *lsl* and *lhx1*b would be required to draw more definitive conclusions (Ganz et al., 2012). Histological sections also revealed expression of *pank2* in ependymal cells at 24 and 48 hpf, and the hydrocephalus we observed in *pank2* morphants could be related to defects in this type of cells and in maintenance of midbrain–hindbrain ventricle structure. Many proteins involved in iron metabolism are expressed in ependymal layer (Benkovic and Connor, 1993, Siddappa et al., 2002) and blood brain barrier (Moos, 2002, McCarthy and Kosman, 2015), where they play an important role in controlling iron transport to and from the brain. As we suggested previously (Poli et al., 2010), defects in PANK2 in these type of cells could result in modification of expression of iron-related proteins, and particularly of ferroportin, the sole cellular iron exporter (Ganz, 2005), and hence shift iron homeostasis in brain toward iron accumulation, one of the main neuropathological feature of PKAN disorder.

Our data also indicate a role of *pank2* in the formation of vessels and maintenance of their integrity since all vascular markers we considered in WISH and the transgenic lines we used in MO experiments indicate severe defects in vascular arborization. In wild-type embryos the caudal vein forms a venous plexus with a reticular pattern (Fig. 9A) and dorsal aorta and cardinal vein are correctly lumenized (Fig. 10C). In most morphants there is a cavernous caudal vein with loss of usual reticular pattern, main axial vessels are fenestrated and the caliber is probably enlarged; moreover, blood flow is severely impaired despite a pumping heart and intervein vessels are largely incomplete, findings consistent with the observed caudal edema and hemorrhages. These observations are completely novel and never documented in existing PKAN animal models or in patients; hence their relevance for the function of the human PANK2 and PKAN pathology is difficult to predict. Our unpublished observations show that *PANK2* is expressed at significant levels in human umbilical vein endothelial (HUVEC) cells. We are currently exploring whether its down-regulation in this cell type could lead to defects in angiogenesis. To this regard, an interesting communication by Hayflick (2006) reported vascular changes in brains from PKAN patients, as detected by histochemistry. If confirmed, this observation would represent an important connection with the phenotype we found in P2-MO-injected fish, and would solicit further investigation of underlying molecular mechanisms.

Since we could observe embryos with clear alteration of the neural structures and limited perturbation of the vascular development, as well as embryos with the opposite features, we speculate that the neural and vascular phenotypes develop in parallel as independent events. This is in accordance with the WISH data showing significant expression of *pank2* gene in both types of tissues. Yet, a better definition of this aspect may only come from investigation of the phenotypes at the molecular level, possibly with the use of transgenic lines targeting specific subsets of cells and molecular pathways.

The phenotype we observed is specific as confirmed by the rescue we obtained when *pank2* mRNA was co-injected with P2-MO. This suggests that, same as in humans and mice, the other gene paralogs (*pank1a*, *pank1b and pank4*) present in zebrafish do not compensate for *pank2* suppression. Very little and only preliminary information is available about these genes in zebrafish: according to WISH data present in the ZFIN database (http://www.zfin.org), during embryonic development *pank1a* is expressed in CNS and retina, while *pank1b* in CNS, retina, and pronephric duct. None of them is present in vascular structures, yet they share with *pank2* the CNS localization. To assess whether down-regulation of *pank2* altered expression levels of these alternate isoforms we quantified the amount of *pank1a* and *pank1b* mRNAs by RT-PCR in ST-MO- and P2-MO-injected embryos at 24 hpf. We did not find any difference in *pank1b* levels at 24 hpf between the two type of samples, while we could not amplify *pank1a* in any sample at 24 hpf, even though the RT-PCR did work with 2 cell-stage embryos. Altogether we can conclude that the phenotype we obtained by *pank2* down-regulation is specific and indicate that difference in function and/or distribution among the existing forms of Pank enzyme preclude significant compensatory effects during zebrafish development. Of great relevance is also the restoration of the wild type phenotype we obtained by exposing P2-MO-injected embryos to 30 μM pantethine. The drug was shown to rescue the phenotype due to PANK2 defects in mammalian cells (Rana et al., 2010, Siudeja et al., 2012), in *Pank2* knock-out mice (Brunetti et al., 2014) and in *fumble* Drosophila mutants and cell lines (Rana et al., 2010, Siudeja et al., 2012). The mechanism of action of pantethine is not clear. Bosveld et al. (2008) suggested that in *Drosophila* it could circumvent the block in CoA biosynthesis due to the lack of phospho-pantothenate, the product of PANK2 activity, being metabolized firstly to pantetheine (Durr and Cortas, 1964, Fisher and Szulc, 1997) and then to phospho-pantetheine, an intermediate of CoA biosynthesis downstream of PANK2 activity. Yet the alternative enzyme catalyzing pantetheine phosphorylation is not known. There is evidence that in humans and rodents pantethine is readily metabolized by pantetheinase to cysteamine and pantothenate (Wittwer et al., 1985, Kaskow et al., 2012), compounds that cannot provide a biochemical bypass for the blockage of CoA biosynthesis. Since cysteamine was able to reproduce the lipid-lowering effect obtained by pantethine administration to human fetal fibroblasts or rabbits and rats (Wittwer et al., 1987) and was shown to have important neuroprotective effects in models of Parkinson's disease (Sun et al., 2010, Gibrat and Cicchetti, 2011) and Huntington's disease (Borrell-Pagès et al., 2006), it is possible that pantethine effect in mice is at least in part mediated by this metabolite. Interestingly, neither cysteamine nor pantothenate could reproduce the increase of about 50% in liver CoA concentration observed in rats treated with pantethine (Wittwer et al., 1987), suggesting the possible existence of multiple mechanisms contributing to the capacity of the drug for correcting defects related to PANK2 malfunctioning. We could not measure CoA levels in the zebrafish embryos and hence have no direct evidence of a link between the phenotype we observed and the shortage of CoA. Nonetheless, the high efficacy of pantethine treatment let infer this interpretation, that is more strongly supported by the result we obtained by addition of CoA to the water of P2-MO injected-embryos. The compound fully prevented the development of the abnormal phenotype, while vitamin B5 was not effective. We speculate that addiction of CoA to water restores normal levels of the metabolite in the embryos and therefore prevents the effects due to the injection of *pank2* morpholino. This is important evidence that the down-regulation of *pank2* in zebrafish alters conserved molecular mechanisms and pathways and hence the model can be useful to both investigate PKAN pathogenesis and perform screening for molecules with therapeutic potential.

## 5 Conclusion

We have investigated the expression and function of *pank2* during early stages of zebrafish development. There is so far no information about the role of this enzyme in vertebrate development and, given the early onset of most PKAN cases, this study could be helpful to understand the disease pathogenesis. Our results suggest that *pank2* downregulation is associated with shortage of CoA in specific sets of cells and tissues, that in turn impairs the development of different regions in the forebrain, and disrupts normal vascular arborization. It will be important to define the subset of affected neural cells, since this could be related to the selective vulnerability of the globus pallidum in PKAN patients. More difficult is to connect the unpredicted role of *pank2* in the formation of vascular structures to PKAN clinical features and pathogenesis, and further work is needed to confirm this aspect in different experimental models. The availability of different transgenic lines for specific signaling cascades (Moro et al., 2013) and the possibility to generate *pank2* knock-out/knock-in models will allow a thorough characterization of the affected cell types and involved biochemical pathways, thus providing essential details for PKAN comprehension and treatment.

The following are the supplementary data related to this article.

**Fig. S2** Zebrafish Pank2 localizes in the cytosol and the nucleus in Cos7 and HeLa cells. HeLa (A, B) and Cos7 (C, D) cells were transfected with the pcDNA3.1-*pank2*-Flag constructs and after 24/48 h stained with anti-Flag antibody, and eventually with DAPI and Mitotracker. In both cell types Pank2-Flag predominantly localizes in the cytosol and nucleus, without significant overlap with the mitochondrial tracer. Size bar = 10 μm in A, B and C; 20 μm in D.
**Fig. S3** WISH with *pank2* sense probe and qRT-PCR analysis of *pank2* expression levels. Digoxigenin-labeled, *pank2* sense probe was synthesized as described in Materials and methods and applied for *in situ* hybridization with embryos at 24 (A) and 48 hpf (B). No specific labeling was evident at any stage. Total RNA was extracted from different developmental stages (C) and adult tissues/organs (C) and analyzed for *pank2* mRNA levels by real time RT-PCR. Results for developmental stages are expressed as relative quantification (RQ) and normalized to *actin beta 1* as endogenous reference gene. This was not possible for adult tissues, because of huge differences in terms of *actin beta 1* and also *eef1a1l1* housekeeping gene levels. In this case, the CT values are presented in the graph; both graphs represent the mean and standard deviation of at least three different experiments.
**Fig. S4** Dose curve of P2-MO. A) To establish the appropriate amount of P2-MO morpholino to be used to suppress *pank2* expression, different doses were injected at the 1/2-cell-stage and the induced mortality evaluated at 24 hpf. The dose of 1 pmol/embryo was chosen as the most effective. The graph shows mean + SD of viable embryos at 24 hpf, expressed as percentage of total injected embryos. B) Representative agarose gel electrophoresis of RT-PCR products for *pank1a* and *pank1b* at the 2-cell stage (control) and at 24 hpf for non-injected and ST-MO- or P2-MO-injected embryos.
**Fig. S5** Heart rate of embryos. The heart rate of embryos was evaluated by direct counting of heart beats per minute (bpm) at 48 hpf in control and P2-MO-injected embryos, eventually treated with CoA or vitamin B5 (VB5). * = P < 0.001. 10 embryos of each type were analyzed in 6 different microinjection experiments.
**Fig. S6***pank2* knock-down does not significantly affect cell proliferation. Phospho-histone H3 (PHH3) staining of proliferating cells in not injected- (B), ST-MO- (C) and pank2 MO (D) embryos (all displayed in full size in A) shows a similar number of positive nuclei (blue spots) in *pank2* morphants, compared to controls. All embryos are at 48 hpf, in lateral view, anterior to the left. Experiments were performed in duplicate, with at least 30 embryos per condition. The chart in E shows the PHH3 cell counting for the left side of the head region in 10 embryos per condition. Although a little decrease of positive cells is detectable in *pank2* morphants, this difference appears not statistically significant.
**Fig. S7** Pantethine dose curve and quantification of its rescue effects. A) Gastrula-stage embryos were exposed to pantethine concentration ranging from 30 to 500 μM and checked for survival at 24 hpf. Doses higher than 50 μM showed significant toxicity. B) The phenotype of ST-MO-, P2-MO- and P2-MO + pantethine 30 μM-exposed embryos was analyzed at 24 hpf. P2-MO injection perturbed normal development in about 70% of injected embryos, but addition of pantethine at gastrula stage largely prevented this effect, allowing normal development in more than 80% of injected embryos.
**Fig. S8** Vitamin B5 does not correct the morphology of embryos injected with P2-MO. Vitamin B5 (VB5) 50 μM was added to fish-water 2 h after injection of P2-MO (C, F), and morphology of embryos compared with that of untreated ones (A, B, D, E) at 48 hpf. The compound does not prevent the appearance of the aberrant phenotype.
**Fig. S9***pank2* knock-down affects vascular morphology and blood cell distribution. Tg(*gata1a*:dsRed) embryos (A, B), with blood cells labeled in red, and Tg(*fli1a*:EGFP) embryos (C, D), with vascular system labeled in green, were micro-injected with ST-MO and P2-MO oligos and analyzed by fluorescence microscopy at 80 hpf. All images are lateral views of the anterior (A, C) or posterior trunk region (B, D). Experiments were performed in duplicate, with at least 30 embryos per condition.
**Table ST1** List of amino acid sequences used for multiple alignment (only PANK2 polypeptides) and phylogenetic analysis.
**Table ST2** List of the primers and their sequences.

## Figures and Tables

**Table 1:** Main morphological alterations observed in P2-MO-injected embryos.

Morphological defects	24 hpf	48 hpf
Altered brain morphology	+++	+++
Edema in hindbrain–midbrain ventricle (hydrocephalus)	+	+++
Edema in the caudal plexus	+	+++
Reduced heart beating rate	+	+++
Reduced blood circulation	+	++

+++ = strong alteration, ++ = moderate alteration, + = mild alteration.
