# Supplementary material for: Knock-down of pantothenate kinase 2 severely affects the development of the nervous and vascular system in zebrafish, providing new insights into PKAN disease
Source: Neurobiol Dis. 2016 Jan;85:35–48. doi: 10.1016/j.nbd.2015.10.010 (PMC4684146; doi:10.1016/j.nbd.2015.10.010)
Supplement: Supplementary file 1 — Fig. S2 Zebrafish Pank2 localizes in the cytosol and the nucleus in Cos7 and HeLa cells. HeLa (A, B) and Cos7 (C, D) cells were transfected with the pcDNA3.1-pank2-Flag constructs and after 24/48 h stained with anti-Flag antibody, and eventually with DAPI and Mitotracker. In both cell types Pank2-Flag predominantly localizes in the cytosol and nucleus, without significant overlap with the mitochondrial tracer. Size bar = 10 μm in A, B and C; 20 μm in D. Fig. S3 WISH with pank2 sense probe and qRT-PCR analysis of pank2 expression levels. Digoxigenin-labeled, pank2 sense probe was synthesized as described in Materials and methods and applied for in situ hybridization with embryos at 24 (A) and 48 hpf (B). No specific labeling was evident at any stage. Total RNA was extracted from different developmental stages (C) and adult tissues/organs (C) and analyzed for pank2 mRNA levels by real time RT-PCR. Results for developmental stages are expressed as relative quantification (RQ) and normalized to actin beta 1 as endogenous reference gene. This was not possible for adult tissues, because of huge differences in terms of actin beta 1 and also eef1a1l1 housekeeping gene levels. In this case, the CT values are presented in the graph; both graphs represent the mean and standard deviation of at least three different experiments. Fig. S4 Dose curve of P2-MO. A) To establish the appropriate amount of P2-MO morpholino to be used to suppress pank2 expression, different doses were injected at the 1/2-cell-stage and the induced mortality evaluated at 24 hpf. The dose of 1 pmol/embryo was chosen as the most effective. The graph shows mean + SD of viable embryos at 24 hpf, expressed as percentage of total injected embryos. B) Representative agarose gel electrophoresis of RT-PCR products for pank1a and pank1b at the 2-cell stage (control) and at 24 hpf for non-injected and ST-MO- or P2-MO-injected embryos. Fig. S5 Heart rate of embryos. The heart rate of embryos was evaluated by direct c [file mmc1.pdf]

S2

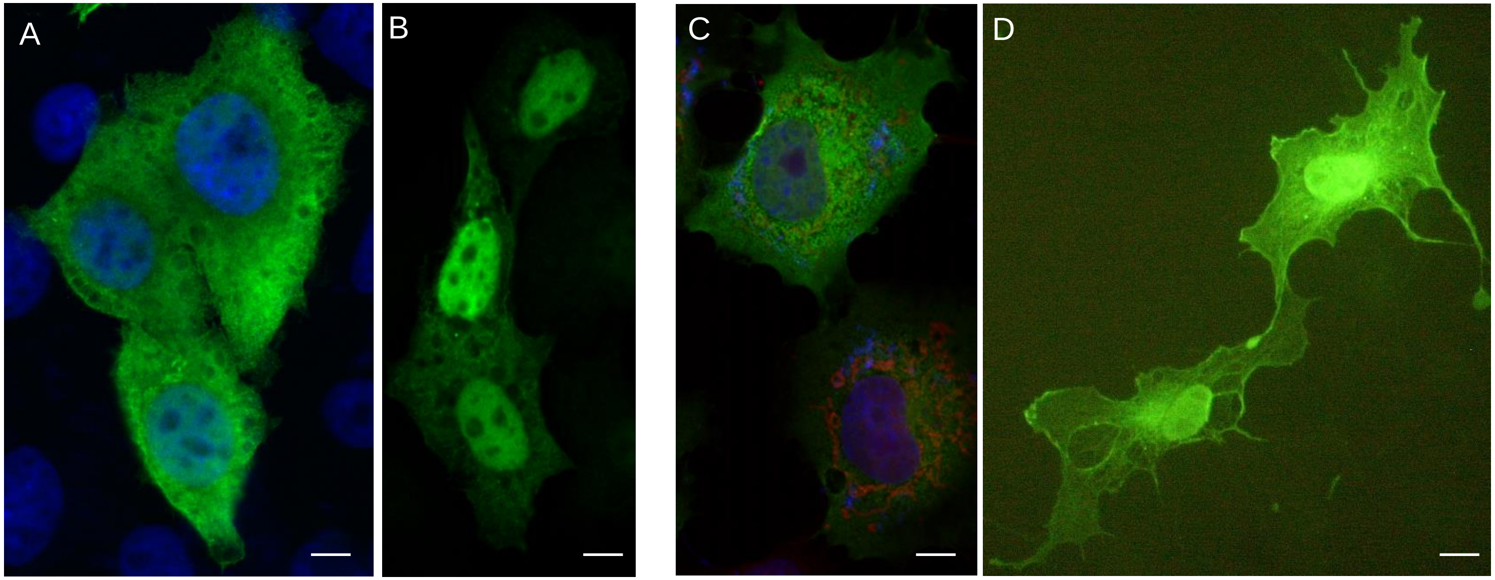

S3

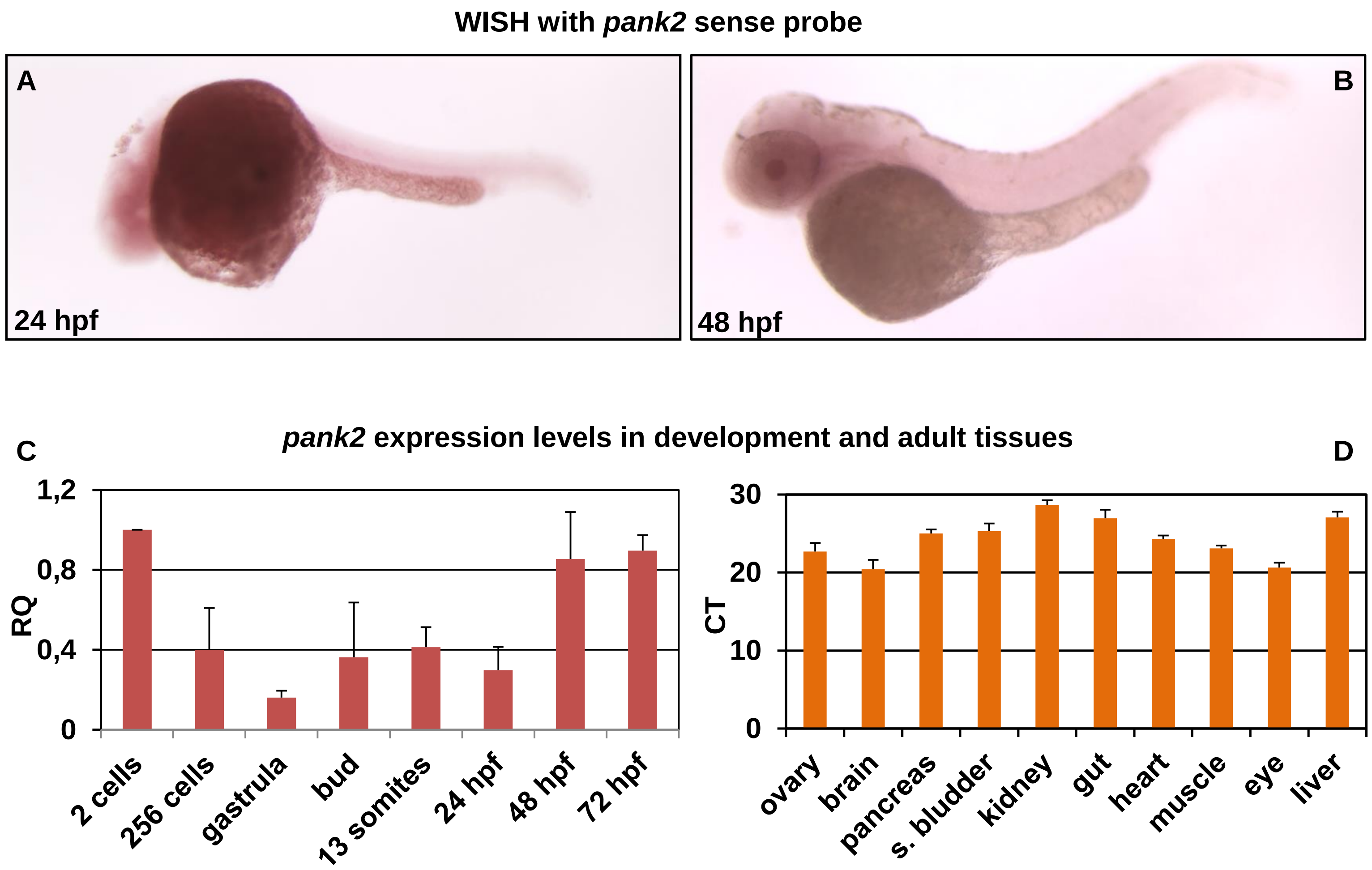
WISH with *pank2* sense probe
A
24 hpf
B
48 hpf
C
pank2 expression levels in development and adult tissues
D
1,2
0,8
0,4
0
RQ
2 cells
256 cells
gastrula
bud
13 somites
24 hpf
48 hpf
72 hpf
30
20
10
0
CT
ovary
brain
pancreas
s. bludder
kidney
gut
heart
muscle
eye
liver

S4

A

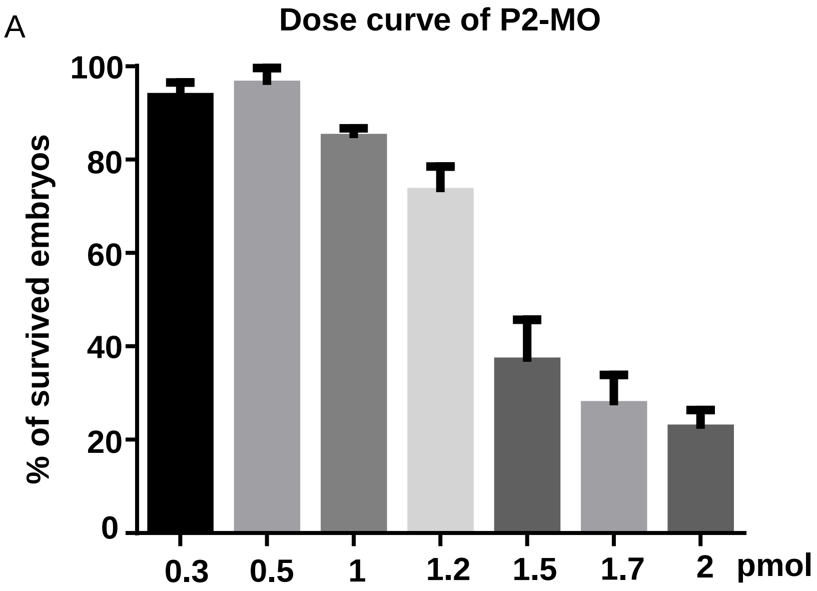

Dose curve of P2-MO
% of survived embryos
100
80
60
40
20
0
0.3
0.5
1
1.2
1.5
1.7
2
pmol

B

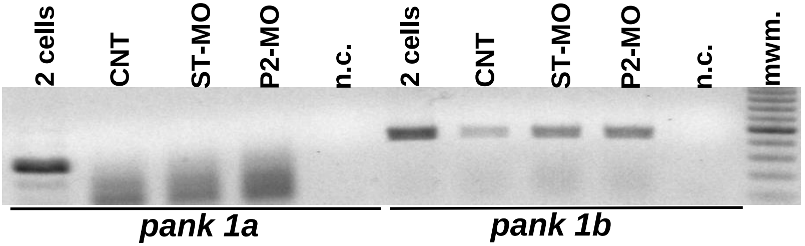

2 cells
CNT
ST-MO
P2-MO
n.c.
2 cells
CNT
ST-MO
P2-MO
n.c.
mwm.
pank 1a
pank 1b

S5

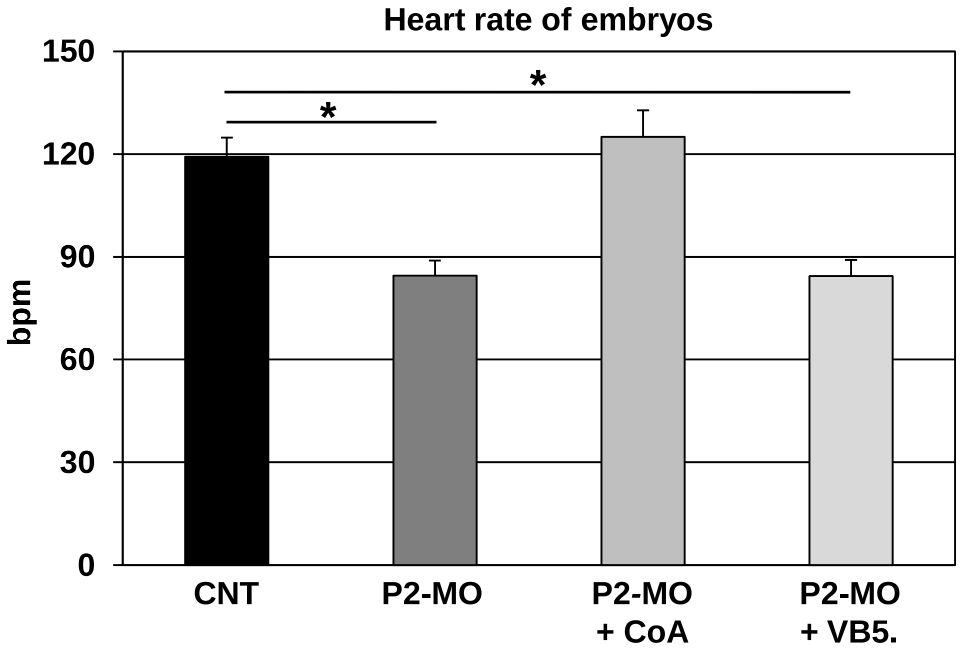

Heart rate of embryos
bpm
150
120
90
60
30
0
*
*
CNT
P2-MO
P2-MO
+ CoA
P2-MO
+ VB5.

S6

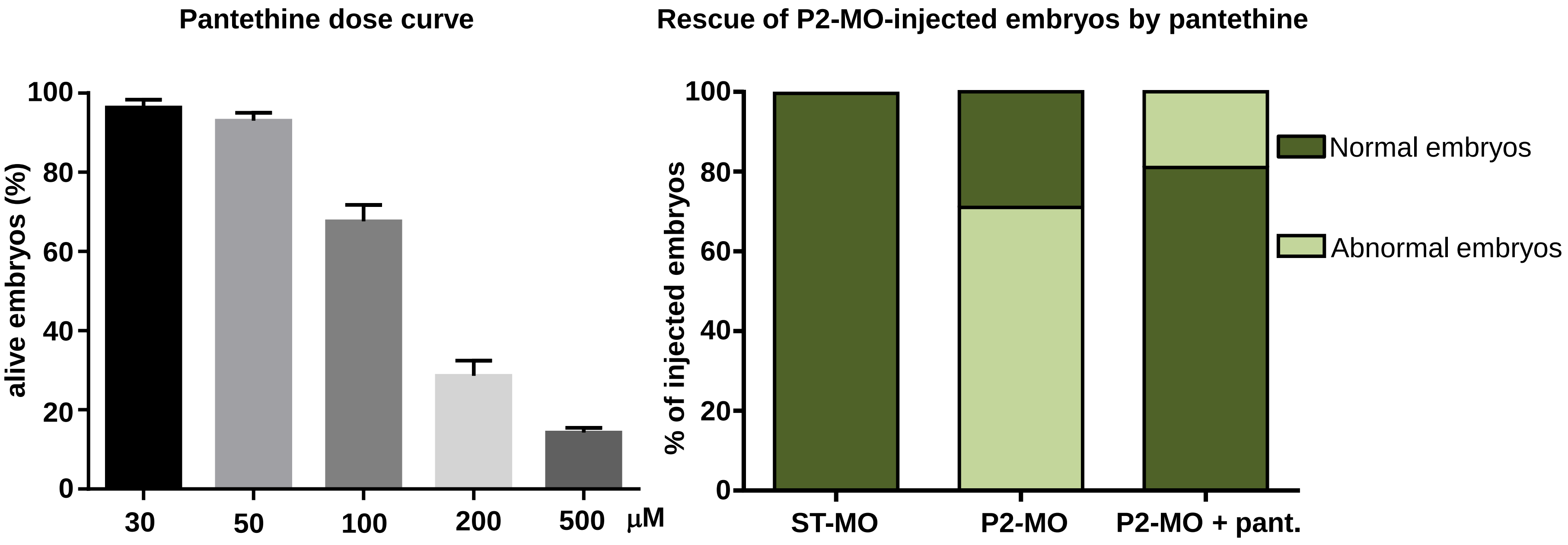
Pantethine dose curve
alive embryos (%)
100
80
60
40
20
0
30
50
100
200
500
µM
Rescue of P2-MO-injected embryos by pantethine
% of injected embryos
100
80
60
40
20
0
ST-MO
P2-MO
P2-MO + pant.
Normal embryos
Abnormal embryos

S7

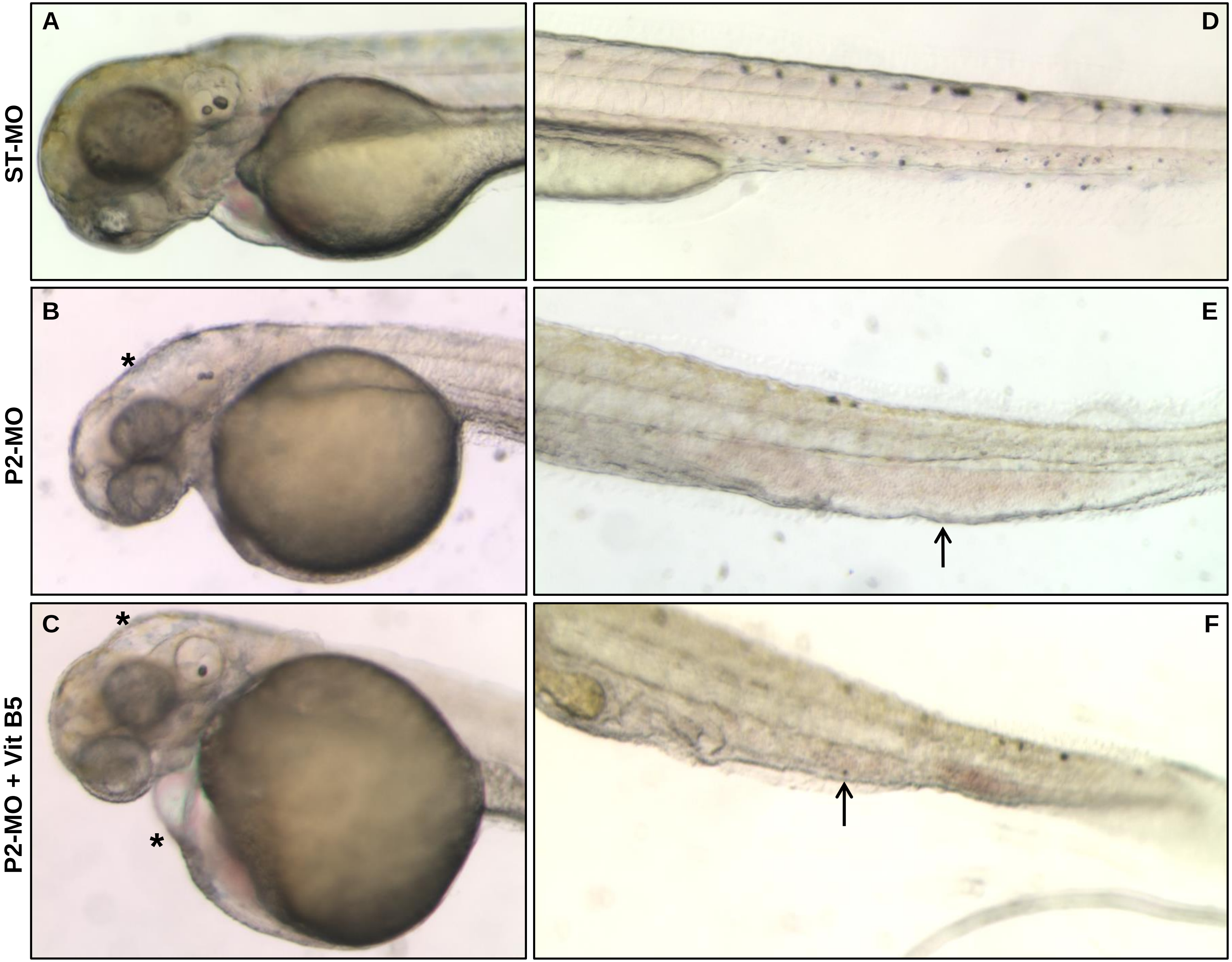

S8

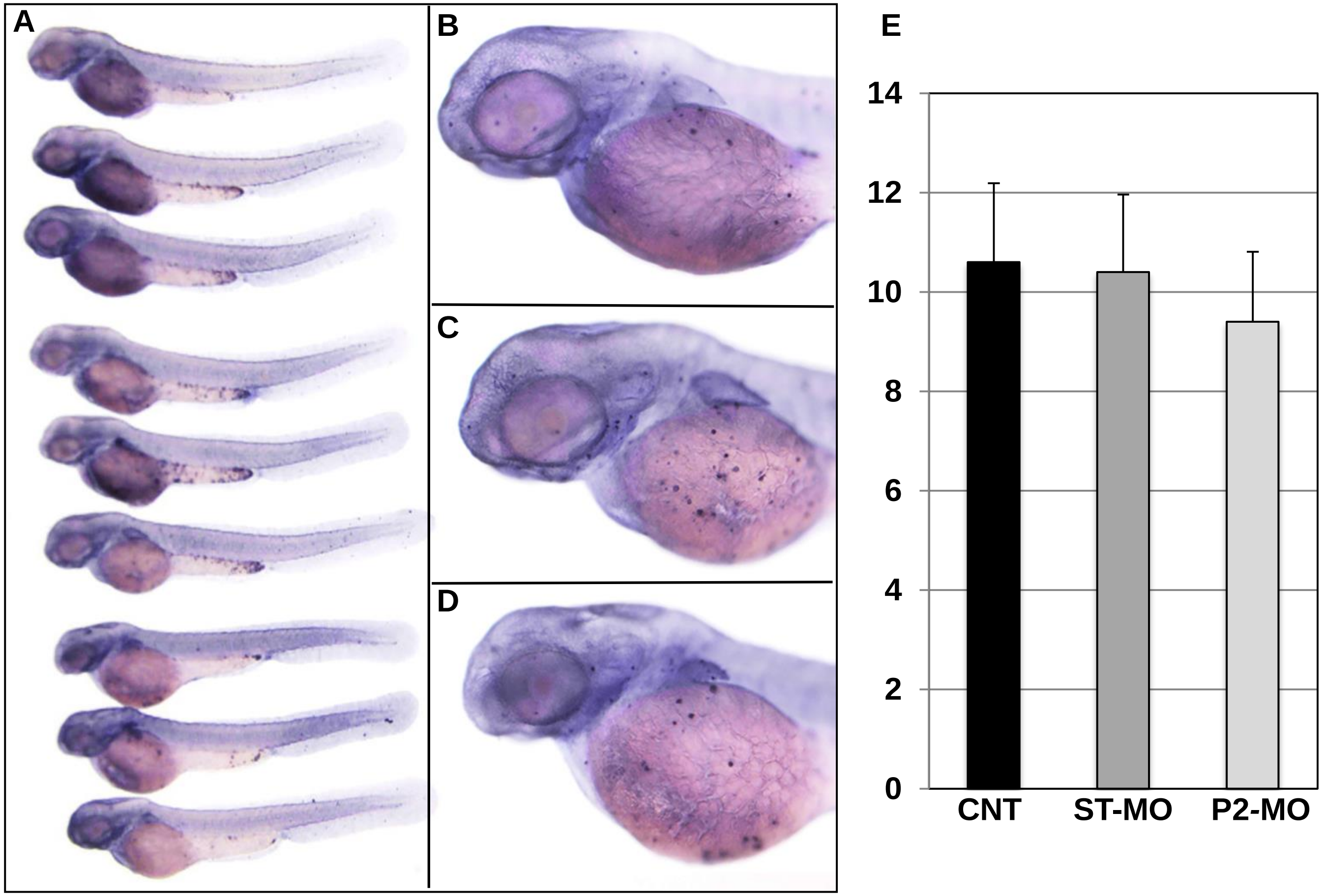

S9

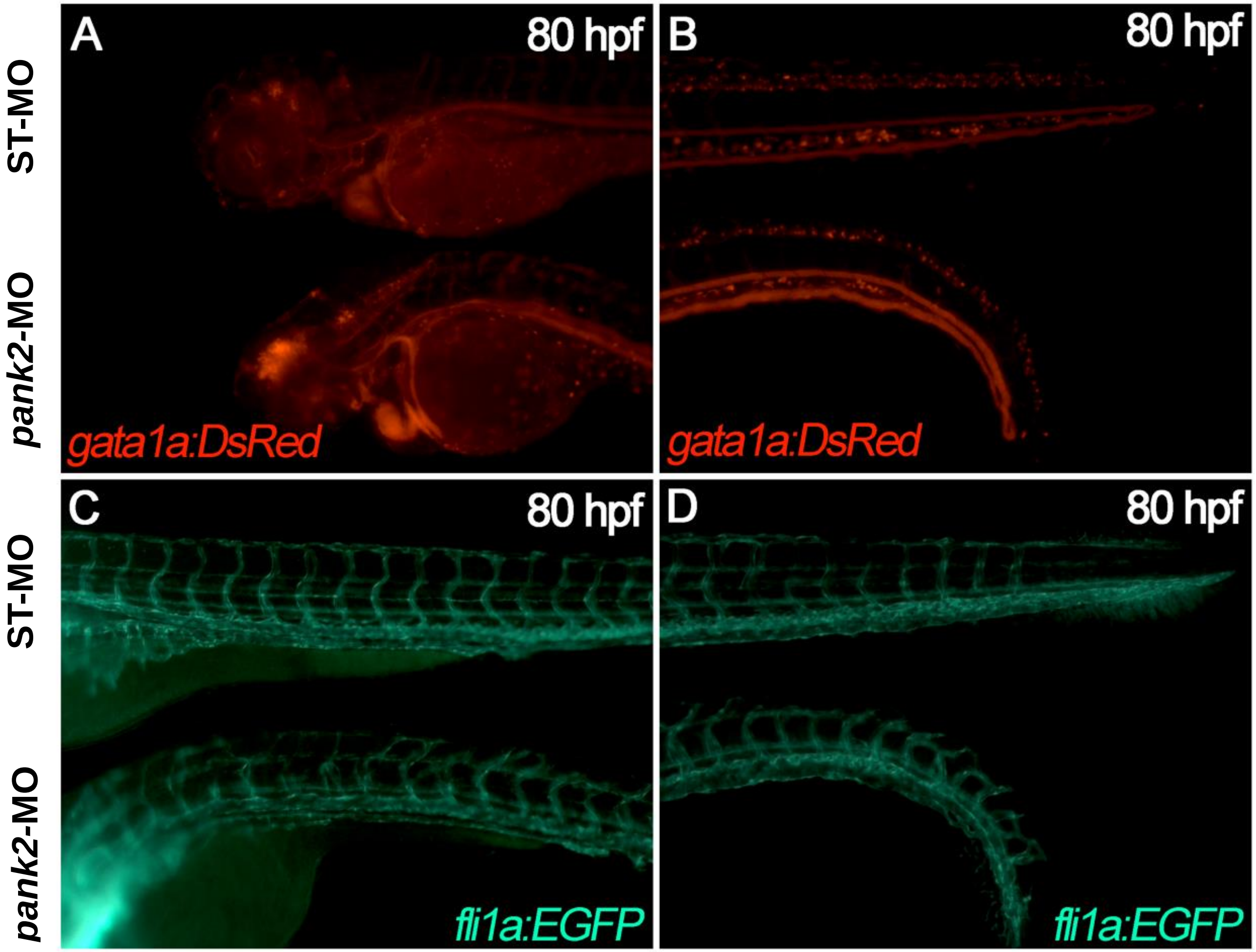
A
80 hpf
B
80 hpf
ST-MO
pank2-MO
gata1a:DsRed
gata1a:DsRed
C
80 hpf
D
80 hpf
ST-MO
pank2-MO
fli1a:EGFP
fli1a:EGFP

ST1

Organism	Abbreviation	Protein	Accession n.
Homo sapiens	Hsa	PANK1	NP_683878
Homo sapiens	Hsa	PANK2	NP_705902
Homo sapiens	Hsa	PANK3	NP_078870
Homo sapiens	Hsa	PANK4	NP_060686
Mus musculus	Mmu	PANK1	NP_001107811
Mus musculus	Mmu	PANK2	NP_705721
Mus musculus	Mmu	PANK3	NP_666074
Mus musculus	Mmu	PANK4	NP_766578
Gallus gallus	Gga	PANK1	XP_421664
Gallus gallus	Gga	PANK2	XP_420873
Gallus gallus	Gga	PANK3	XP_414502
Gallus gallus	Gga	PANK4	NP_001231841
Danio rerio	Dre	Pank1a	NP_998645
Danio rerio	Dre	Pank1b	NP_001077310
Danio rerio	Dre	Pank2	NP_001074075
Danio rerio	Dre	Pank4	NP_956809
Drosophila melanogaster	Dme	fumble	NP_524870
Tricomonas vaginalis	Tva	fumble	XP_001290292

Table ST1. List of amino acid sequences used for multiple alignment (only PANK2 polypeptides) and phylogenetic analysis.

ST2

Name	Primer sequence (5'→3')
P2-MO	TAACAAGATGGTTCACCTACGCGGC
ST-MO	CCTCTTACCTCAGTTACAATTTATA
P1	TCCATGAAGTTGCTCGCGTA
P2	CAGCTCAGGATGGGTTTGGT
P3	ACCTGCTAGCATGGAGCTGAACGGCTTC
P4	ACCTGGATCCTCACTTGTCATCGTCATCCTTGTAATCGGATGG GTTTGGTAGTTCCAA
P5	ATGGAGCTGAACGGCTTCC
P6	TCAGGATGGGTTTGGTAGTTCC
P7	GCTGGATGAGCTGGAGTGTT
P8	AACACTCCAGCTCATCCAGC
P9	ACCTGGATCCACCATGGAGCTGAACGGCTTC
P10	ACCTTCTAGATCAGGATGGGTTTGGTAGTTC
P11	CGAGGGATGTACGGAGAAGC
P12	GCCAATGTTGACAAGCAGCA
A1	CGAGCAGGAGATGGGAACC
A2	CAACGGAAACGCTCATTGC
EF1	GAGAAGTTCGAGAAGGAAGC
EF2	CGTAGTATTTGCTGGTCTCG

Table ST2. List of the primers and their sequences.
